# Tirzepatide attenuates mesolimbic cocaine-evoked dopamine levels and reduces cocaine taking, motivation and seeking behaviours in male rodents

**DOI:** 10.1016/j.ebiom.2026.106219

**Published:** 2026-03-23

**Authors:** Christian E. Edvardsson, Xinming Zhang, Thaynnam A. Emous, Louise Adermark, Sarah Witley, Mia Ericson, Heath D. Schmidt, Elisabet Jerlhag

**Affiliations:** a Department of Pharmacology, Institute of Neuroscience and Physiology, The Sahlgrenska Academy, University of Gothenburg, Gothenburg, Sweden; b Department of Biobehavioral Health Sciences, School of Nursing, University of Pennsylvania, Philadelphia, PA, USA; c Department of Psychiatry, Perelman School of Medicine, University of Pennsylvania, Philadelphia, PA, USA; d Department of Psychobiology, Paulista School of Medicine (EPM), Federal University of São Paulo (UNIFESP), Sao Paulo, Brazil; e Addiction Biology Unit, Department of Psychiatry and Neurochemistry, Institute of Neuroscience and Physiology, The Sahlgrenska Academy, University of Gothenburg, Gothenburg, Sweden

**Keywords:** GLP-1, GIP, Incretin, Cocaine, Addiction, Dopamine, GABA, Glutamate

## Abstract

**Background:**

Cocaine use disorder (CUD) remains among the most treatment-resistant addictions, characterised by high relapse rates even following extended abstinence periods. Dopamine signalling in mesocorticolimbic circuits contributes to cocaine's reinforcing effects and remains an important target for therapeutic intervention. Growing evidence suggests appetite-regulating peptides may influence central dopamine transmission. Whether recently approved incretin polyagonists can modulate cocaine-related behaviours through their capacity to simultaneously engage multiple appetite-regulating peptide receptor pathways remains unexplored.

**Methods:**

Here we investigated whether tirzepatide, a clinically approved long-acting dual glucose-dependent insulinotropic polypeptide (GIP)/glucagon-like peptide 1 (GLP-1) receptor agonist, alters cocaine-related behavioural and neurochemical responses in male rodents.

**Findings:**

We found that tirzepatide produced dose-dependent reductions in cocaine self-administration (P < 0.001) and diminished cocaine-evoked dopamine responses, as evidenced by attenuated locomotor stimulation (P < 0.001), conditioned place preference (P < 0.001), and accumbal dopamine levels (P < 0.01) across two cocaine doses. Tirzepatide also reduced the motivation to self-administer cocaine (P < 0.05) and attenuated the reinstatement of cocaine-seeking behaviour in animal models of relapse (P < 0.001). Beyond these effects, tirzepatide prevented the expression of cocaine-induced locomotor sensitisation (P < 0.001), suggesting it may have broader neuroadaptive effects. Neurochemical analyses revealed that tirzepatide normalised cocaine-induced dopamine elevations in mesocorticolimbic circuits (P < 0.001) and the lateral septum (P < 0.001), consistent with the behavioural attenuation and our accumbal microdialysis data. We also observed effects on GABA (P < 0.01) and glutamate (P < 0.01) signalling in these regions, suggesting possible multi-neurotransmitter mechanisms. Principal component analysis indicated that tirzepatide affects neural substrates regulating mesocorticolimbic function, potentially contributing to the observed effects on accumbal dopamine release.

**Interpretation:**

When considering our findings alongside tirzepatide's clinical availability, the evidence suggests this incretin polyagonist merits investigation as a potential treatment approach for CUD.

**Funding:**

The study is supported by grants (EJ) from the 10.13039/501100004359Swedish Research Council (2023-2600 and 2025-07154), LUA/ALF (grant no. 723941) from the 10.13039/501100005754Sahlgrenska University Hospital, Alcohol Research Council of the Swedish Alcohol Retailing Monopoly (FO2024-0048), 10.13039/501100014552Adlerbertska Research Foundation (2024-791), Wilhelm & Martina Lundgren’s Research Foundation (2024-SA-4698) and 10.13039/501100014197Mary von Sydow Foundation (2024-36). This work was also supported by the following grants from the 10.13039/100000002National Institutes of Health (NIH): R01 DA037897 and R01 DA061799 (H.D.S.) Thaynnam A Emous held an international internship scholarship from the São Paulo Research Foundation (FAPESP), Process Number #2023/18470-5, while conducting research at the University of Gothenburg.

**Research in context**

**Evidence before this study**

Cocaine use disorder (CUD) continues to pose significant global health challenges, with persistent drug cravings during abstinence contributing to high relapse rates. The absence of approved pharmacotherapies for individuals seeking treatment reflects a substantial unmet medical need. Cocaine acts through well-characterised mesocorticolimbic dopamine circuits that drive drug taking, reward processing, and seeking behaviours, yet translating this mechanistic knowledge into clinical interventions has proven elusive. Emerging evidence suggests that modulating interconnected neural circuits through appetite-regulating peptides may offer alternative approaches where direct dopamine pathway targeting has yielded limited success. Among these candidates, glucagon-like peptide-1 receptor (GLP-1R) agonists have shown promise in preclinical studies, effectively modifying cocaine self-administration, conditioned place preference, and reinstatement behaviours. The recent development of incretin polyagonists presents a potential advancement in the addiction field. Tirzepatide, a dual glucose-dependent insulinotropic polypeptide receptor (GIPR) and GLP-1R agonist, favours GIPR over GLP-1R binding while preserving distinct GLP-1R signalling properties. This profile appears relevant given emerging evidence that GIPR may influence addiction processes both independently and synergistically with GLP-1R. Despite clinical approval for type 2 diabetes and obesity, tirzepatide's effects on stimulant drugs like cocaine remain largely unexplored. This knowledge gap warrants attention given the potential for multi-target engagement in treating the complex neuroadaptations that characterise substance use disorders.

**Added value of this study**

Here we provide a systematic investigation of tirzepatide's effects across multiple dimensions of cocaine addiction liability in male rodents. Our work demonstrates that tirzepatide produces dose-dependent reductions in cocaine self-administration and diminishes the motivation to obtain cocaine under progressive ratio testing. Beyond voluntary drug taking, tirzepatide attenuates cocaine-evoked dopamine responses, as evidenced by reduced locomotor stimulation, conditioned place preference, and accumbal dopamine levels measured via microdialysis. Importantly, tirzepatide blocks the reinstatement of cocaine-seeking behaviour across species and paradigms, suggesting effects on relapse vulnerability. Tirzepatide also prevents expression of cocaine-induced locomotor sensitisation, indicating potential influence on neuroadaptive processes. Neurochemical analyses reveal that tirzepatide normalises cocaine-induced alterations in dopamine, GABA, and glutamate across mesocorticolimbic circuits and the lateral septum. These effects occur without signs of malaise or general behavioural suppression.

**Implications of all the available evidence**

Given tirzepatide's established clinical approval and availability, these findings position this incretin dual agonist as a candidate worthy of further investigation for CUD treatment. The consistent findings across behavioural paradigms, neurochemical measures, and multiple species suggests tirzepatide may address several dimensions of CUD, from voluntary drug taking to relapse vulnerability. When considered alongside emerging clinical evidence that GLP-1R agonists reduce alcohol consumption in humans, these preclinical findings support further investigation of tirzepatide as a potential pharmacotherapy for a disorder that currently lacks any approved treatment options.

## Introduction

Cocaine use disorder (CUD) continues to pose significant challenges globally,^1^^,^^2^ with persistent drug cravings during abstinence, indicative of a dysfunctional dopamine system, as a contributing factor to relapse.^3^, ^4^, ^5^, ^6^, ^7^ Yet no approved pharmacotherapies currently exist for individuals seeking treatment.^1^^,^^2^^,^^8^ Previous research has indicated a central role for dopamine in cocaine taking, reward-related, and drug-seeking behaviours.^6^^,^^9^ Direct targeting of mesocorticolimbic dopamine pathways, however, has yielded limited therapeutic success. More effective interventions may arise by targeting interconnected neural circuits and pathways that contribute to CUD, particularly those involving dopamine signalling, motivational drive, and relapse vulnerability.^6^^,^^10^^,^^11^

The gut–brain axis provides a salient example of such network-level modulation, as several substrates have been identified as candidates for treating substance use disorders, including CUD,^12^, ^13^, ^14^, ^15^, ^16^ partly due to their capacity to act on multiple therapeutically relevant targets simultaneously.^15^^,^^17^ Particularly interesting are the incretin hormones glucagon-like peptide-1 (GLP-1) and glucose-dependent insulinotropic polypeptide (GIP), which combine well-characterised metabolic roles^13^^,^^17^ with emerging evidence for dopamine system modulation.^12^^,^^14^^,^^15^ Preclinical evidence shows GLP-1 receptor (GLP-1R) agonists effectively modify cocaine self-administration, conditioned place preference (CPP), and reinstatement behaviours,^18^, ^19^, ^20^, ^21^, ^22^, ^23^, ^24^, ^25^, ^26^, ^27^ while the role of GIP receptor (GIPR) signalling in these same paradigms has received no attention.

Recent pharmaceutical advances have produced polyreceptor agonists that simultaneously engage multiple appetite-regulating peptide targets.^17^^,^^28^, ^29^, ^30^, ^31^ Among these developments, tirzepatide stands as a long-acting dual GIPR/GLP-1R agonist and the first of its kind to achieve approval for type 2 diabetes and obesity management.^28^, ^29^, ^30^, ^31^ Interestingly, tirzepatide favours GIPR over GLP-1R binding while preserving distinct GLP-1R signalling properties,^32^ potentially relevant since GIPR seems to influence addiction processes independently and in combination with GLP-1R.^33^ Clinically, tirzepatide has demonstrated favourable therapeutic outcomes for metabolic disorders and fewer side effects compared to GLP-1R monotherapies.^28^, ^29^, ^30^, ^31^ These findings point towards tirzepatide as a potentially promising approach for substance use disorders, where complex neuroadaptations may benefit from interventions that might engage multiple pathways concurrently. Indeed, tirzepatide has been shown to reduce alcohol intake and relapse-like behaviours in rodents,^34^ yet whether these effects extend to cocaine and the mesocorticolimbic mechanisms underlying such actions remains unclear.

Here, we investigated whether tirzepatide influences cocaine-related behaviours across multiple dimensions of addiction liability. We examined its effects on self-administration and reward-related responses, assessed motivational changes through progressive ratio testing, and explored relapse-like responses via reinstatement paradigms in both operant and place preference models. Additionally, cocaine-induced locomotor sensitisation allowed us to assess tirzepatide's impact on neurotransmitter adaptations in several brain regions following repeated cocaine exposure. We focused on two fundamental questions: whether tirzepatide affects cocaine-mediated behaviours and, if so, what underlying neurotransmitter alterations within addiction-relevant brain circuits might contribute to these behavioural changes.

## Methods

### Animals

Adult male NMRI mice (n = 310, 8–10 weeks, 25–30 g upon arrival; Charles River, Sulzfeld, Germany) and adult male Sprague–Dawley rats (n = 42, 7 weeks of age, 225–250 g at arrival; Taconic Laboratories, Rensselaer, NY, USA and Ejby, Denmark) served as subjects. Mice were used for locomotor and exploration tests, CPP, and microdialysis studies, while rats were employed for self-administration and locomotor experiments. Species and strain were based on established protocols from our previous investigations.^25^^,^^34^^,^^35^ Only male animals were used in this study. Although sex differences in cocaine responses and the GLP-1 system have been documented preclinically,^34^, ^35^, ^36^, ^37^, ^38^, ^39^, ^40^, ^41^ and sex-dependent effect to cocaine are recognised clinically,^37^^,^^42^ the present study did not include females due to resource and experimental design constraints. This limitation is addressed more fully in the Discussion. Following arrival, animals underwent a one-week acclimatisation period in group under standardised housing conditions: 12:12-h light/dark cycle, 20 °C ambient temperature, and 50% relative humidity. Food (standard chow; Purina LabDiet 5001 for rats; Harlan Teklad Rodent Diet #2916 for mice) and water were provided *ad libitum*. Housing configurations differed by experimental requirements: self-administration rats remained individually housed throughout self-administration procedures to prevent catheter damage and enable individual intake measurements, while mice were group-housed except for post-surgical microdialysis subjects, which required individual housing to protect implants. All subjects were handled on at least three occasions before behavioural testing. Experimental procedures were conducted during the light phase. Animals were randomised to equal sized balanced treatment groups. Researchers involved in assessing experimental data were blinded to the treatment conditions. A between-subjects design was employed for most experiments, except for the self-administration and reinstatement experiments (Exp 9–11), which utilised a within-subjects counterbalanced design. Each experiment was conducted once, except the self-administration and reinstatement paradigms, which was conducted within two independent cohorts, though practical constraints (our capacity to test only 4–6 animals simultaneously in certain protocols) necessitated distributing testing across multiple groups per day over several days. Results remained consistent across all groups and days. A total of 18 rats and 34 mice underwent surgical procedures. Animals were excluded according to pre-established criteria: technical complications (catheter patency loss n = 2 rats; probe misplacement n = 4 mice) or animal illness (unrelated to surgery or treatment, n = 1 rat).

### Ethics

All experiments received approval from the Ethics Committee for Animal Research in Gothenburg, Sweden (ethical permits: 4685/23 and 3348/20) or the Institutional Animal Care and Use Committee (University of Pennsylvania; protocol #805705). Studies adhered to the NIH Guide for the Care and Use of Laboratory Animals, ARRIVE guidelines, and 3Rs principle.

### Drugs

Tirzepatide (LY3298176 hydrochloride, MedChemExpress, Sollentuna, Sweden) was dissolved in vehicle (40 mM Tris–HCl with 0.01% Tween, pH 8.0) and administered subcutaneously (SC) 30 min (mice) or 90 min (rats) before behavioural testing. Dose selection (3, 30 and 70 nmol/kg) was based on our previous tirzepatide-alcohol study^34^ where neither of the two higher doses affected general behaviour. An additional lower dose (3 nmol/kg) was tested in locomotor and self-administration paradigms to explore the effect of lower boundary of tirzepatide's dose–response profile. The extended pretreatment interval in rats was selected to ensure adequate drug exposure during behavioural assessment, as our previous study^34^ demonstrated minimal effects on 4-h intake when tirzepatide was administered 30 min before alcohol access. Tirzepatide exhibits an approximate half-life of 12 h in rodents,^43^^,^^44^ substantially shorter than in humans (>5 days),^44^ though effects persisted beyond 24 h but not 48 h.^34^ These differing pretreatment intervals likely reflect interspecies variation in pharmacokinetic or pharmacodynamic profiles that warrants further investigation. To verify that tirzepatide's effect on cocaine-related responses did not result from motor impairment or non-specific behavioural suppression, we first tested the 30 and 70 nmol/kg doses on exploratory and anxiety-related behaviours (open field, elevated plus maze and light–dark box tests) in mice (n = 36, 12 per group), described in Supplementary Information. Spontaneous locomotor activity was also assessed in rats (n = 24, 6 per group) using all three doses (3, 30 or 70 nmol/kg) or vehicle. Neither dose in either species altered motor function or induced general behavioural effects (Supplementary Figures S1 and S2). Cocaine hydrochloride (National Institute on Drug Abuse, Rockville, MD, USA; Apoteket AB, Gothenburg, Sweden) was dissolved in vehicle (0.9% NaCl; saline). Cocaine was always administered intraperitoneally (IP) at 10 or 20 mg/kg, 5 min before behavioural testing. In self-administration studies, cocaine was delivered intravenously 0.245 mg/kg per infusion. Doses were based on our previous investigations.^18^^,^^25^^,^^26^ Modified Ringer's solution (140 mM NaCl, 1.2 mM CaCl_2_, 3.0 mM KCl, 1.0 mM MgCl_2_; Sigma–Aldrich, Darmstadt, Germany) served as the perfusion solution for the microdialysis experiments.

### Locomotor activity and locomotor sensitisation in mice

Locomotor activity was recorded in sound-attenuated, dimly lit (3 lx) locomotor boxes (42 × 42 × 20 cm; Open Field Activity System, Med Associates) using infrared photobeam detection (Activity Monitor software, Version 7, Med Associates).^34^^,^^35^ For all locomotor stimulation and sensitisation experiment test days, mice (n = 36, 9 per group in each experiment) first underwent 60-min habituation to the test arena, received vehicle or tirzepatide treatment, then received vehicle or cocaine injection 30 min later. Activity recording commenced 5 min after the final injection and continued for 60 min. In Exp 1–4, we examined tirzepatide (3, 30 or 70 nmol/kg) effects on acute cocaine (10 or 20 mg/kg)-induced locomotor stimulation, which reflects mesolimbic system activation.^45^ In Exp 13, we investigated whether tirzepatide (30 nmol/kg) could suppress the expression of behavioural locomotor sensitisation to cocaine (10 mg/kg), the enhanced locomotor response following repeated drug exposure.^11^ For the sensitisation paradigm, mice received vehicle or cocaine on day 1 during locomotor testing, followed by daily home cage injections (days 2–8) to induce sensitisation. After a two-day withdrawal period (days 9–10), mice underwent challenge testing on day 11 with vehicle or tirzepatide pretreatment followed by vehicle or cocaine. Upon completion of Exp 13, brains were rapidly collected and stored at −80 °C for neurochemical analysis.

### Conditioned place preference—expression and reinstatement in mice

CPP experiments utilised two-chambered arenas (50 × 24 × 24 cm, custom-made, University of Gothenburg) with distinct tactile and visual cues under dim lighting (3 lx).^34^^,^^35^ Following a 20-min pretest (day 1) to assess initial place preference mice underwent conditioning sessions (days 2–5) using a biased design pairing cocaine with the least preferred chamber and vehicle with the preferred chamber. Daily conditioning included one cocaine and one vehicle session, alternating morning and afternoon. In Exp 5 and 6 (n = 20 per experiment, 10 per group) mice received cocaine conditioning (10 mg/kg in Exp 5; 20 mg/kg in Exp 6). On day 6, mice received vehicle or tirzepatide (30 nmol/kg) pretreatment 30 min before the 20-min expression test. In Exp 12 (n = 20, 10 per group), we investigated tirzepatide's effects on cocaine-primed reinstatement. After identical pretest (day 1), conditioning (day 2–5, cocaine 10 mg/kg), mice were received vehicle on the expression test (day 6), to confirm place conditioning. Cocaine preference was then extinguished (days 7–10) through twice-daily vehicle pairings with both chambers. Extinction criteria (<5% of group average expression score) was confirmed on day 11, and mice were divided into balanced groups based on preference scores. On day 12, mice received vehicle or tirzepatide (30 nmol/kg) 30 min before a cocaine (10 mg/kg) priming injection, followed by the 20-min reinstatement test. Behaviour were analysed with Observer XT software (Version 15, Noldus, Wageningen, Netherlands). CPP was quantified as percentage change in drug-paired compartment time between pre-test and test sessions.

### Microdialysis in mice

*In vivo* microdialysis was conducted in mice (n = 34 initally, 4 excluded, 16–18 per experiment, 8–9 per group) across two separate experiments (Exp 7 and 8) to investigate tirzepatide's effects on cocaine-evoked accumbal dopamine transmission. I-shaped microdialysis probes (20 kDa cut-off membrane, 1 mm active length; HOSPAL, Gambro, Lund, Sweden) were surgically implanted in the nucleus accumbens (NAc) shell four days before experimentation, as previously described.^18^^,^^34^^,^^35^ On experiment days, probes were perfused with Ringer's solution at 1.6 μl/min. Following a 2-h equilibration, microdialysate samples were collected at 20-min intervals. Baseline measurements were obtained (minutes −40 to 0), followed by tirzepatide (30 nmol/kg) or vehicle administration (minute 10) and cocaine injection (Exp 7: 10 mg/kg, Exp 8: 20 mg/kg) 30 min later (minute 40), with nine additional post-cocaine injection samples collected. Probe placement was verified histologically *post mortem* using a brain atlas.^46^ Only correctly positioned probes without haemorrhage were analysed (Supplementary Figure S3A). Dialysate samples were analysed using HPLC with electrochemical detection.^35^ Changes in monoamine levels were calculated as percentages of the mean of three baseline values before treatment. Area under the curve (AUC) following cocaine exposure (40-220 min) was calculated to assess overall treatment effects.

### Operant self-administration in rats

To explore tirzepatide's effects on voluntary cocaine taking (self-administration; Exp 9), motivation to self-administer cocaine (progressive ratio; PR; Exp 10) and cocaine seeking during abstinence (reinstatement; Exp 11), we employed operant self-administration in rats (n = 18, initally, 3 excluded). These paradigms model key components of CUD,^2^^,^^4^^,^^6^^,^^7^ Rats were tested across two independent cohorts using a within-subjects, counterbalanced design. The first cohort (n = 8) was tested for cocaine taking and reinstatement, while the second cohort (n = 10) was tested for cocaine taking, motivation and reinstatement.

### Catheterisation surgery

Prior to surgery, rats were anaesthetised with ketamine (100 mg/kg; Covetrus, Portland, ME, USA) and 10 mg/kg xylazine (Covetrus). An indwelling catheter (SAI Infusion Technologies, Lake Villa, IL, USA) was inserted into the right jugular vein and sutured in place. The catheter was routed subcutaneously to a mesh backmount that was implanted above the shoulder blades. To prevent infection and maintain patency, catheters were flushed daily with 0.2 ml of the antibiotic Timentin (0.93 mg/ml; Fisher, Pittsburgh, PA, USA) dissolved in heparinised 0.9% NaCl (Butler Schein, Dublin, OH, USA). When not in use, catheters were sealed with plastic obturators.

### Cocaine self-administration

Rats were allowed seven days to recover from surgery before behavioural testing commenced. Initially, rats were placed in operant conditioning chambers (Med Associates Inc., Georgia, VT, USA) and allowed to lever-press for intravenous cocaine infusions (0.245 mg/kg per infusion, delivered over more than 5 s) on a fixed ratio 1 (FR1) schedule of reinforcement, consistent with our previous studies.^22^^,^^25^^,^^27^ Rats could self-administer a maximum of 30 infusions during each daily 2-h operant session. Once a rat achieved at least 15 infusions in a single session under the FR1, it was advanced to a fixed ratio 3 (FR3) schedule. Following similar criterion achievement under FR3 (>15 infusions), rats progressed to a fixed ratio (FR5) schedule. For all FR schedules, a 20-s timeout period followed each cocaine infusion, during which active lever presses were recorded but had no scheduled consequences. Inactive lever presses, which had no scheduled consequences, were also recorded. Following 21 days of cocaine self-administration, rats were tested using a within-subjects, counterbalanced design (n = 15). Vehicle or tirzepatide (3 or 30 nmol/kg) was administered 90 min before FR5 test session (Exp 9). Between test days, rats self-administered cocaine on the FR5 schedule to ensure responding had stabilised and drug treatment had washed out. After completing FR5 testing, a subset of rats (n = 8) was switched to a PR schedule of reinforcement to assess motivation to self-administer cocaine (Exp 10). Under the PR schedule, the response requirement for each subsequent infusion increased exponentially according to the equation R(i) = [5e^0.2i^−5] until the rat failed to meet a requirement within 60 min.^27^^,^^47^^,^^48^ Breakpoint was operationally defined as the last response requirement completed before session termination. Rats received vehicle or tirzepatide (30 nmol/kg) 90 min before PR test sessions in a within-subjects, counterbalanced design. Between PR test days, rats self-administered cocaine on the FR5 schedule to maintain stable baseline responding.

### Cocaine reinstatement

Following completion of the self-administration phase, drug-taking behaviour was extinguished by replacing the cocaine with saline. Daily extinction sessions continued until active lever responding fell below 20% of the total active lever responses completed on the final day of cocaine self-administration. For the 15 rats used in reinstatement studies, mean (±standard error of the mean (SEM)) active lever responses on the last self-administration day were 137.4 ± 5.4, which decreased to 26.9 ± 1.1 upon meeting extinction criteria (typically 5-7 days). Once cocaine self-administration was extinguished, rats entered the reinstatement phase. To reinstate cocaine-seeking behaviour, rats received an acute priming injection of cocaine (10 mg/kg, IP) immediately before a 2-h test session. During reinstatement sessions, every fifth lever press resulted in a saline infusion. Using a within-subjects, counterbalanced design, rats (n = 15) received vehicle or tirzepatide (3 or 30 nmol/kg) 90 min before the cocaine priming injection subsequent reinstatement test (Exp 11). Each reinstatement test was followed by extinction sessions until responding again fell below 20% of the final self-administration day (generally 1 and 2 days).

### Ad libitum food and kaolin intake

Rats (n = 15) were housed in hanging wire cages throughout the self-administration experiment to monitor food intake and assess potential malaise-like effects in cocaine-experienced animals, consistent with our previous GLP-1R agonist studies.^47^^,^^49^ Following test sessions, rats had *ad libitum* access to standard chow. Food spillage was collected on papers beneath cages, and intake was calculated as the difference in hopper weights minus spillage. Given GLP-1R agonists can produce nausea and emesis in drug-naïve rodents and humans^50^^,^^51^ we measured kaolin clay consumption as an indicator of pica behaviour, the consumption of non-nutritive substances in response to emetic agents.^47^^,^^49^^,^^52^ Rats were habituated to kaolin availability for seven days before testing. Both food and kaolin consumption were assessed 24 h post-tirzepatide administration.

### Neurochemical tissue analysis of monoamines and amino acids

Given that tirzepatide suppressed the expression of cocaine-induced sensitisation, brain tissue from Exp 13 was analysed for neurotransmitter concentrations across multiple brain regions to explore potential neurochemical substrates. Brains were maintained on ice during processing and sectioned using a brain matrix. Regions of interest, all previously implicated in addiction neurocircuitry,^10^^,^^53^ were microdissected using brain punching needles. Tissue samples were collected from the NAc, ventral tegmental area (VTA), prefrontal cortex (PFC), lateral septum (LS), dorsolateral striatum (DLS), dorsomedial striatum (DMS), and amygdala (AMY) as previously described.^34^^,^^35^ Samples were weighed and stored at −80 °C until analysis. On analysis days, tissue samples were homogenised and the tissue-free supernatant was analysed using HPLC with either electrochemical detection for monoamines (noradrenaline, dopamine, serotonin, and their metabolites normetanephrine (NM), 3-methoxytyramine (3-MT), 3,4-dihydroxyphenylacetic acid (DOPAC), 5-hydroxyindoleacetic acid (5-HIAA), and homovanillic acid (HVA)) or with fluorescent detection for amino acids (glutamate, γ-aminobutyric acid (GABA), glycine, taurine, serine and glutamine) as described previously^35^^,^^54^^,^^55^ and in Supplementary Information. External standards were used for peak identification and quantification, with all values normalised to tissue sample weight.

### Statistics

Statistical analyses were performed using GraphPad Prism (version 10.5.0, GraphPad Software Inc., Boston, MA, USA) following the same approach as previously described.^25^^,^^34^^,^^35^ Data normality was assessed using the Shapiro–Wilk test and Q–Q plots, and all statistical tests employed two-tailed significance testing with α = 0.05. Sample sizes were determined based on our prior tirzepatide-alcohol work, where n = 7–12 reliably detected medium to large effect sizes (e.g., d = 1.73 for CPP with n = 10),^34^ and informed by earlier GLP-1R agonist studies in cocaine models that produced more modest effect sizes (d ≈ 0.6–0.8) alongside considerable inter-individual variability. Given uncertainty about tirzepatide's dual GIPR/GLP-1R agonism in cocaine paradigms, which characteristically show greater response variability than alcohol models, we used n = 7–10 for between-subjects experiments (locomotor activity, CPP, microdialysis) to maintain adequate power, accounting for 10-20% exclusions from misplaced microdialysis probes. Self-administration studies employed a within-subjects counterbalanced design across two independent cohorts (n = 8 and n = 10; total n = 18 starting animals), as each subject served as its own control, reducing the number of animals required. Running two cohorts enabled replication while accommodating technical attrition including catheter complications and allowed verification that effects were not cohort-specific. Statistical approaches were matched to experimental design: unpaired, paired, or one-sample t-tests for two-group comparisons; two-way ANOVAs (treatment × cocaine) with Bonferroni post-hoc tests for locomotor activity experiments; paired t-tests or repeated-measures one-way ANOVAs (one-way) with Bonferroni post-hoc corrections for self-administration, microdialysis (treatment × time), and locomotor sensitisation (treatment × day) data; and one-way ANOVAs with Bonferroni post-hoc tests (comparing all groups) for *ex vivo* tissue neurochemistry. Multivariate analyses of neurochemical data employed Pearson's correlation and principal component analysis (PCA), with group differences in principal component scores assessed by unpaired t-tests (two-group comparisons) or one-way ANOVAs with Bonferroni post-hoc tests (four-group comparisons). Data are presented as mean ± SEM with individual data points displayed where appropriate.

### Role of funders

The funding sources did not play a role for the study design, collection, analysis, and interpretation.

## Results

### Tirzepatide dose-dependently attenuates cocaine dopamine-related behaviours and cocaine-evoked changes in accumbal dopamine in mice

As cocaine's dopamine-stimulating properties underpin much of its addiction liability,^6^^,^^10^^,^^56^^,^^57^ we sought to determine whether tirzepatide could modulate cocaine-induced dopamine-related responses across multiple behavioural and neurochemical paradigms.

**Fig. 1:**
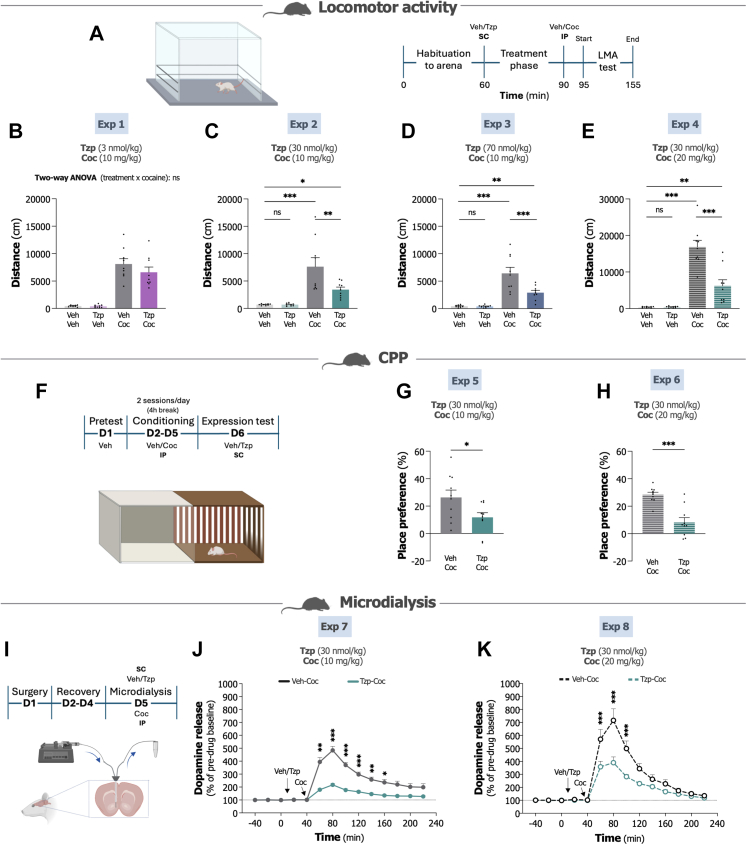
**Tirzepatide dose-dependently attenuates cocaine-induced dopamine-related behaviours, and attenuates cocaine-elevated accumbal dopamine levels in mice. A.** Experimental outline for locomotor activity (LMA) tests. **B.** While the lowest dose (3 nmol/kg) of tirzepatide (Tzp) did not significantly block cocaine (Coc; 10 mg/kg)-induced locomotor stimulation (n = 9/group, two-way ANOVA (treatment × cocaine)). **C and D.** Higher doses (30 and 70 nmol/kg) of tirzepatide significantly attenuate cocaine (10 mg/kg)-induced locomotor stimulation (n = 9/group, two-way ANOVA (treatment × cocaine) followed by Bonferroni post hoc test). **E.** The 30 nmol/kg dose of tirzepatide also mitigates cocaine-induced locomotor stimulation with a higher cocaine dose (20 mg/kg) (n = 9/group, two-way ANOVA (treatment × cocaine) followed by Bonferroni post hoc test). **F.** Experimental outline for conditioned place preference (CPP) experiments. **G.** Tirzepatide (30 nmol/kg) reduces the expression of cocaine (10 mg/kg) CPP compared to vehicle (n = 10/group, unpaired t-test). **H.** Tirzepatide (30 nmol/kg) also decreases the expression of cocaine (20 mg/kg) CPP compared to vehicle (n = 10/group, unpaired t-test). **I.** Experimental outline for the microdialysis studies. **J.** Tirzepatide (30 nmol/kg) attenuates cocaine (10 mg/kg)-evoked dopamine levels in NAc shell compared to vehicle controls (n = 8/group, repeated measures two-way ANOVA (time × treatment) followed by Bonferroni post hoc test). **K.** Tirzepatide (30 nmol/kg) attenuates cocaine (20 mg/kg)-evoked dopamine levels in NAc shell compared to vehicle-treated mice (n = 7/group, repeated measures two-way ANOVA (time × treatment) followed by Bonferroni post hoc test). Data are presented as mean ± SEM. ns; non-significant, ∗P < 0.05, ∗∗P < 0.01, ∗∗∗P < 0.001.

Our initial approach tested three doses of tirzepatide (3, 30 and 70 nmol/kg) against cocaine-induced locomotor stimulation at both a low (10 mg/kg) and high (20 mg/kg) dose (experimental outline in Fig. 1A). In Exp 1, the lowest tirzepatide dose (3 nmol/kg) did not alter cocaine-induced locomotor stimulation (treatment F_1,32_ = 1.31, P = 0.261; cocaine F_1,32_ = 105.00, P < 0.001; interaction treatment × cocaine F_1,32_ = 1.17, P = 0.288, two-way ANOVA; Fig. 1B). Increasing the dose to 30 nmol/kg in Exp 2 revealed clearer efficacy. Tirzepatide alone again had no impact on baseline locomotion (P > 0.999, Bonferroni), and cocaine (10 mg/kg) once more elicited substantial locomotor stimulation (P < 0.001, Bonferroni) (treatment F_1,32_ = 6.03, P = 0.020; cocaine F_1,32_ = 32.06, P < 0.001; interaction treatment × cocaine F_1,32_ = 6.07, P = 0.019, two-way ANOVA; Fig. 1C). At this dose, tirzepatide pretreatment notably attenuated cocaine-induced hyperlocomotion (P = 0.009, Bonferroni), however locomotor activity levels were still higher than vehicle controls (P = 0.031, Bonferroni). The highest dose (70 nmol/kg) in Exp 3 showed similar attenuation. Baseline locomotion with tirzepatide alone remained unaffected (P > 0.999, Bonferroni), whereas cocaine (10 mg/kg) continued producing marked stimulation (P < 0.001, Bonferroni) (treatment F_1,32_ = 9.03, P = 0.005; cocaine F_1,32_ = 51.57, P < 0.001; interaction treatment × cocaine F_1,32_ = 8.70, P = 0.006, two-way ANOVA; Fig. 1D). Tirzepatide pretreatment significantly blunted cocaine's hyperlocomotor effects (P < 0.001, Bonferroni), though activity levels stayed elevated relative to vehicle (P = 0.032, Bonferroni). To test whether the effective 30 nmol/kg dose could attenuate a more robust cocaine challenge, Exp 4 employed 20 mg/kg cocaine. Tirzepatide alone did not affect baseline locomotion (P > 0.999, Bonferroni), while cocaine elicited pronounced locomotor stimulation (P < 0.001, Bonferroni) (treatment F_1,32_ = 18.25, P < 0.001; cocaine F_1,32_ = 79.99, P < 0.001; interaction treatment × cocaine F_1,32_ = 18.38, P < 0.001, two-way ANOVA; Fig. 1E). Tirzepatide pretreatment substantially reduced cocaine-induced hyperlocomotion (P < 0.001, Bonferroni), though activity remained elevated compared to vehicle (P = 0.015, Bonferroni).

We next evaluated whether tirzepatide (30 nmol/kg) could influence place preference for cocaine, testing both low (10 mg/kg) and high (20 mg/kg) doses in separate experiments (experimental outline in Fig. 1F). In Exp 5, vehicle-treated mice developed significant preference for the cocaine (10 mg/kg)-paired chamber (P < 0.001, one-sample t-test), yet tirzepatide pretreatment attenuated this conditioned response (t_18_ = 2.29, P = 0.034, unpaired t-test; Fig. 1G). This attenuation proved even more pronounced with the higher cocaine dose in Exp 6. Vehicle-treated mice again displayed robust preference for the cocaine (20 mg/kg)-paired chamber (P < 0.001, one-sample t-test), which tirzepatide markedly reduced (t_18_ = 5.20, P < 0.001, unpaired t-test; Fig. 1H).

To investigate the neurochemical mechanisms involved in these behavioural effects, we employed *in vivo* microdialysis to measure tirzepatide's (30 nmol/kg) impact on cocaine-evoked dopamine release in the NAc shell, testing both 10 and 20 mg/kg cocaine (experimental outline in Fig. 1I). In Exp 7, cocaine (10 mg/kg) markedly elevated accumbal dopamine concentrations, an effect tirzepatide substantially attenuated (treatment F_1,14_ = 56.80, P < 0.001; time F_13,182_ = 76.82, P < 0.001; interaction treatment × time F_13,182_ = 23.13, P < 0.001, repeated measures two-way ANOVA; Fig. 1J). Tirzepatide similarly dampened cocaine-evoked dopamine with the higher dose in Exp 8. Here, cocaine (20 mg/kg) produced robust dopamine elevation, which tirzepatide again reduced (treatment F_1,12_ = 9.38, P = 0.010; time F_13,156_ = 65.30, P < 0.001; interaction treatment × time F_13,156_ = 7.79, P < 0.001, repeated measures two-way ANOVA; Fig. 1K). Additional monoamine graphs and AUC analyses for both experiments appear in Supplementary Figures S4 and S5.

These convergent findings across behavioural and neurochemical paradigms indicate that tirzepatide attenuates cocaine's reinforcing effects, potentially through modulation of mesolimbic dopamine signalling.

### Tirzepatide reduces cocaine self-administration and the motivation to self-administer cocaine in rats

**Fig. 2:**
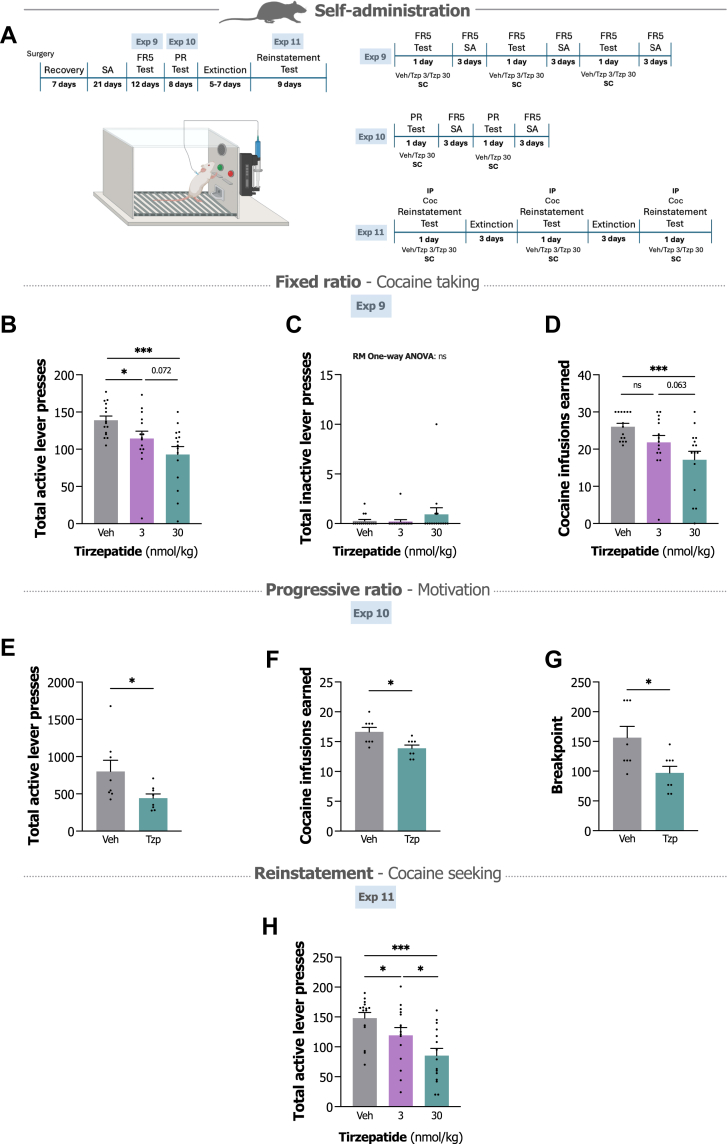
**Tirzepatide reduces cocaine self-administration, motivation to self-administer cocaine and reinstatement in rats. A.** Experimental timeline depicts the cocaine self-administration (SA) paradigm with fixed ratio 5 (FR5), progressive ratio (PR) and reinstatement test phases outlined. Using a within-subjects counterbalanced design, rats were pretreated with either vehicle or tirzepatide (Tzp; 3 or 30 nmol/kg) during the test days. **B and C.** Tirzepatide dose-dependently reduces active lever presses during the FR5 test, while inactive lever responding remains unchanged (n = 15/group, repeated measures one-way ANOVA followed by Bonferroni post hoc test). **D.** Total number of earned cocaine infusions are significantly reduced by the 30 nmol/kg tirzepatide dose (n = 15/group, repeated measures one-way ANOVA followed by Bonferroni post hoc test). **E.** Tirzepatide (30 nmol/kg) reduces active lever presses during the PR test (n = 8/group, paired t-test). **F and G.** Total number of earned cocaine infusions and breakpoint are significantly reduced by tirzepatide (30 nmol/kg) (n = 8/group, paired t-test). **H.** Tirzepatide (3 or 30 nmol/kg) dose-dependently reduces active lever presses during the cocaine-primed (Coc; 10 mg/kg) reinstatement test (n = 15/group, repeated measures one-way ANOVA followed by Bonferroni post hoc test). Data are presented as mean ± SEM. ns; non-significant, ∗P < 0.05, ∗∗P < 0.01, ∗∗∗P < 0.001.

Having observed tirzepatide's capacity to attenuate dopamine-related processes in mice, we next examined whether these effects translated to voluntary cocaine taking in rats. Experimental outlines for Exp 9–11 are presented in Fig. 2A.

In Exp 9, rats received vehicle or tirzepatide (3 or 30 nmol/kg) before FR5 self-administration test sessions. Tirzepatide markedly reduced active lever responding in a dose-dependent manner (treatment F_2,28_ = 13.10, P < 0.001, repeated measures one-way ANOVA; Fig. 2B). Both the 3 nmol/kg (P = 0.032, Bonferroni) and 30 nmol/kg (P < 0.001, Bonferroni) doses decreased active lever pressing relative to vehicle. Inactive lever responding remained unaffected (repeated measures one-way ANOVA; Fig. 2C). This dose-dependent suppression extended to earned cocaine infusions (F_2,28_ = 10.81, P < 0.001, repeated measures one-way ANOVA; Fig. 2D), with the 30 nmol/kg dose significantly decreasing total number of cocaine infusions versus vehicle (P < 0.001, Bonferroni).

Given that GIPR/GLP-1R agonists influence feeding and body weight, we additionally measured 24-h home cage food intake and body weight following test sessions. The higher tirzepatide dose reduced food intake compared to both vehicle (P < 0.001, Bonferroni) and the 3 nmol/kg dose (P < 0.001, Bonferroni) (F_2,28_ = 72.00, P < 0.001, repeated measures one-way ANOVA; Supplementary Figure S6A), and similarly decreased body weight relative to controls (P < 0.001, Bonferroni) (F_2,28_ = 72.00, P < 0.001, repeated measures one-way ANOVA; Supplementary Figure S6B). Importantly, tirzepatide did not increase kaolin intake (a measure of nausea/malaise) in cocaine-experienced rats (F_2,28_ = 0.31, P = 0.735, repeated measures one-way ANOVA; Supplementary Figure S6C). The lower dose had no significant effects on any of these parameters.

To assess whether tirzepatide affects the motivation to self-administer cocaine, we tested the 30 nmol/kg dose in rats responding on a PR schedule (Exp 10). Tirzepatide reduced active lever pressing compared to vehicle (t_7_ = 2.38, P = 0.049, paired t-test; Fig. 2E), while leaving inactive responding unchanged. Both cocaine infusions earned (t_7_ = 3.27, P = 0.014, paired t-test; Fig. 2E) and breakpoint (t_7_ = 2.95, P = 0.022, paired t-test; Fig. 2F) decreased under tirzepatide treatment. There were no effects of treatment on FR5 baseline responding following test days (treatment F_2,14_ = 0.23, P = 0.801, repeated measures one-way ANOVA; Supplementary Figure S7A and treatment F_2,14_ = 0.72, P = 0.523, repeated measures one-way ANOVA; Supplementary Figure S7B).

The dose-dependent suppression of voluntary cocaine taking, coupled with the absence of kaolin consumption, suggests tirzepatide reduces cocaine intake through mechanisms unlikely to involve malaise or aversive visceral effects. The reductions in active lever responding, infusions and breakpoint under a PR schedule of reinforcement further indicate that tirzepatide may specifically alter the motivational salience of cocaine, rather than simply impairing motor function or inducing non-specific behavioural suppression.

### Tirzepatide attenuates the reinstatement of cocaine-seeking behaviour in rats and the reinstatement of place preference in mice

Given that mesolimbic dopamine signalling is implicated in relapse risk,^4^^,^^5^^,^^10^^,^^11^^,^^57^ we employed two complementary paradigms to examine whether tirzepatide could suppress relapse-like behaviours across species.

In Exp 11, we first assessed tirzepatide's (3 or 30 nmol/kg) capacity to block drug-primed reinstatement of cocaine seeking in rats following extinction. Rats underwent extinction sessions until active lever responding fell below 20% of their final self-administration session baseline (final self-administration session: 137.4 ± 5.4 active responses; extinction criterion met at 26.9 ± 1.1 active responses). Following successful extinction, a priming injection of cocaine (10 mg/kg) robustly reinstated lever pressing in vehicle-treated rats. Tirzepatide treatment dose-dependently reduced this reinstatement (treatment F_2,28_ = 10.58, P < 0.001, repeated measures one-way ANOVA; Fig. 2G). Both the 3 nmol/kg (P = 0.044, Bonferroni) and 30 nmol/kg (P < 0.001, Bonferroni) doses significantly attenuated active lever responding relative to vehicle, with the higher dose producing greater suppression (P = 0.038, Bonferroni). Inactive lever responding remained unaffected. There were no statistical differences in drug seeking in the vehicle-treated rats across test days (test day F_2,8_ = 0.22, P = 0.811, repeated measures one-way ANOVA; Supplementary Figure S7C).

**Fig. 3:**
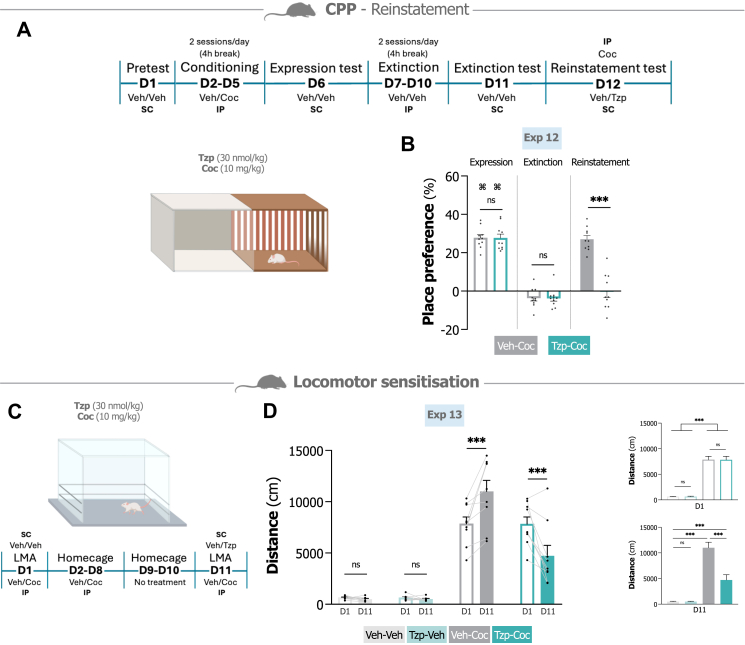
**Tirzepatide blocks reinstatement of place preference and the expression of cocaine-induced locomotor sensitisation in mice. A.** Experimental outline for the conditioned place preference (CPP) reinstatement test. On the reinstatement test day, mice were pretreated with tirzepatide (Tzp; 30 nmol/kg) or vehicle 30 min before receiving a priming dose of cocaine (Coc; 10 mg/kg) just before being placed in the arena. **B.** Reinstatement of place preference is blocked by tirzepatide treatment compared to vehicle (n = 10/group, unpaired t-test). **C.** Experimental timeline for the locomotor sensitisation paradigm. **D.** Cocaine (10 mg/kg) induces locomotor sensitisation in vehicle-cocaine-treated mice, this effect is blocked by tirzepatide (30 nmol/kg) (n = 9/group, repeated measures two-way ANOVA (day × treatment) followed by Bonferroni post hoc test). Data are presented as mean ± SEM. ns; non-significant, ∗P < 0.05, ∗∗P < 0.01, ∗∗∗P < 0.001, **^⌘^**P < 0.001 versus pretest (one sample t-test).

We next examined whether tirzepatide (30 nmol/kg) could similarly prevent cocaine-primed reinstatement in the CPP model (experimental outline in Fig. 3A). In Exp 12, mice underwent cocaine (10 mg/kg) CPP followed by extinction training. During the initial expression test, both treatment groups displayed significant preference for the cocaine-paired environment (P < 0.001, one-sample t-test), which was subsequently extinguished (P > 0.999, one-sample t-test). Following a priming injection of cocaine, vehicle-treated mice exhibited robust reinstatement of place preference (P < 0.001, one-sample t-test). Tirzepatide pretreatment blocked this reinstatement response (t_18_ = 7.68, P < 0.001, unpaired t-test; Fig. 3B).

The consistent suppression of cocaine-primed reinstatement across distinct species and behavioural paradigms suggests tirzepatide may modulate neural substrates involved in relapse-related processes.

### Tirzepatide mitigates cocaine-induced locomotor sensitisation in mice

Locomotor sensitisation following repeated drug exposure is thought to reflect addiction-relevant neuroadaptations, potentially sharing neural substrates with craving and relapse vulnerability.^7^^,^^11^ We therefore examined whether tirzepatide (30 nmol/kg) could suppress the expression of cocaine (10 mg/kg)-induced locomotor sensitisation in mice (Exp 13) (experimental outline in Fig. 3C).

The analysis revealed significant effects (treatment F_3,32_ = 63.80, P < 0.001; day F_1,32_ = 0.04, P = 0.850; interaction treatment × day F_3,32_ = 18.56, P < 0.001, repeated measures two-way ANOVA; Fig. 3D). Repeated cocaine administration produced robust sensitisation, as evidenced by increased locomotor responses in vehicle-cocaine-treated mice between day 1 and day 11 (P < 0.001, Bonferroni). Acute tirzepatide pretreatment on the challenge day effectively blocked expression of this sensitised response, reducing locomotor activity to levels even below those observed during initial cocaine exposure on day 1 (P < 0.001, Bonferroni). When comparing treatment groups on the challenge day (day 11), cocaine induced substantial locomotor stimulation relative to vehicle (P < 0.001, Bonferroni), an effect tirzepatide markedly attenuated (P < 0.001, Bonferroni). Activity levels in tirzepatide-cocaine-treated mice remained elevated compared to vehicle controls (P < 0.001, Bonferroni). Tirzepatide administered alone did not statistically differ from vehicle (P > 0.999, Bonferroni).

The capacity to suppress behavioural sensitisation expression suggests tirzepatide may interfere with neuroadaptive processes that emerge from repeated cocaine exposure.

### Tirzepatide modulates neurochemical alterations induced by repeated cocaine exposure in a brain region-specific manner

As tirzepatide prevented cocaine-induced behavioural sensitisation (Exp 13), we further examined tissue concentrations of monoamines and amino acids in addiction-relevant brain regions from these same mice to explore potential neurochemical substrates. We focused primarily on dopamine, GABA, and glutamate in the NAc, VTA, PFC, and LS given their established roles in addiction-related dopaminergic processes.^10^^,^^53^ Data for additional neurotransmitters and brain regions appear in Supplementary Figures S8–S12.

**Fig. 4:**
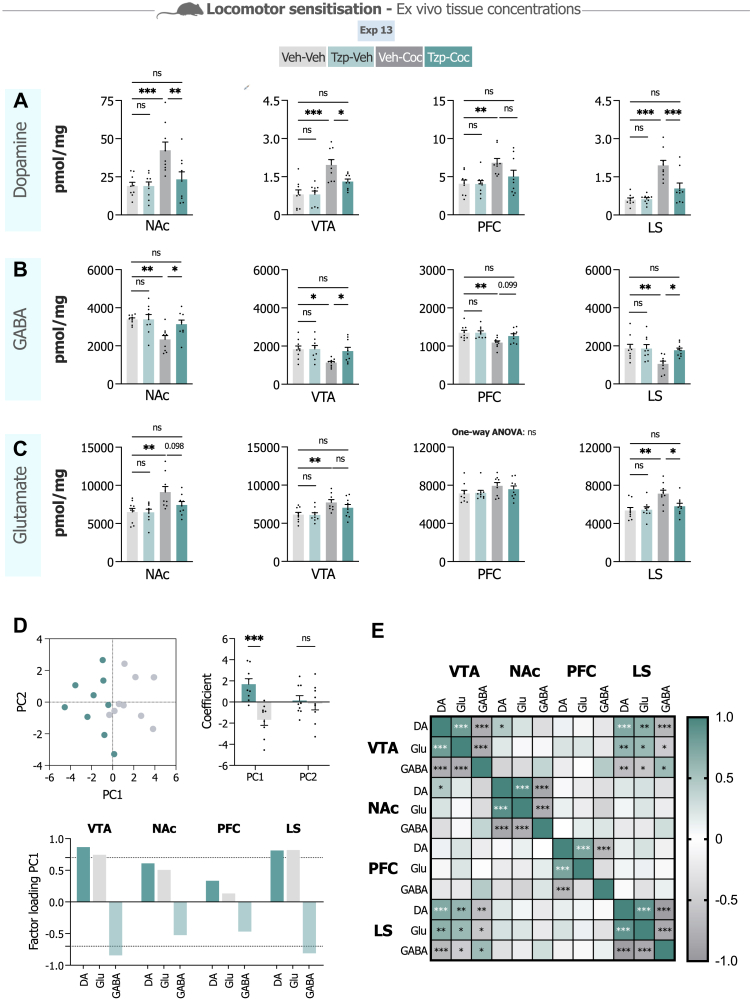
**Dopamine, GABA and glutamate alterations across the mesocorticolimbic circuits and lateral septum following tirzepatide treatment in cocaine-sensitised mice. A–C.** Ex vivo tissue concentration analysis of dopamine (DA), GABA and glutamate (Glu) in cocaine-sensitised mice across nucleus accumbens (NAc), ventral tegmental area (VTA), prefrontal cortex (PFC) and the lateral septum (LS) following repeated cocaine (10 mg/kg) or vehicle exposure and either tirzepatide (Tzp; 30 nmol/kg) or vehicle treatment administered on the last test day (n = 9/group, one-way ANOVA followed by Bonferroni post hoc test). **A.** Dopamine concentrations show cocaine-induced alterations that tirzepatide normalises in the NAc, VTA, and LS, but not the PFC. **B.** GABA concentrations were significantly depressed by cocaine but restored by tirzepatide in the NAc, VTA, and LS. **C.** Glutamate levels were enhanced by cocaine in all brain regions except the PFC. The increase in glutamate was significantly reduced by tirzepatide in the LS. **D.** A principal component analysis (PCA), based on the two cocaine-exposed groups, demonstrated that PC1 could distinguish between subjects and that neurotransmitters in the VTA and LS strongly correlated with the underlying principal component. Dotted line marks a factor loading of 0.7 (unpaired t-test). **E.** Correlation matrix based on Pearson correlation coefficients demonstrated strong interregional associations between neurotransmitters and a correlation between the VTA and LS. Data are presented as mean ± SEM. ns; non-significant, ∗P < 0.05, ∗∗P < 0.01, ∗∗∗P < 0.001.

Dopamine analysis revealed region-specific alterations across multiple brain circuits (Fig. 4A). In the NAc, cocaine elevated dopamine concentrations (P < 0.001, Bonferroni) (F_3,32_ = 7.54, P < 0.001, one-way ANOVA), an increase tirzepatide pretreatment reversed (P = 0.007, Bonferroni), restoring levels towards vehicle values (P > 0.999, Bonferroni). The VTA showed comparable cocaine-induced elevations (P < 0.001, Bonferroni) (F_3,32_ = 11.64, P < 0.001, one-way ANOVA) that tirzepatide attenuated (P = 0.022, Bonferroni), bringing concentrations closer to baseline (P = 0.098, Bonferroni). The PFC presented a similar pattern, although with less statistical significant differences. While cocaine increased PFC dopamine (P = 0.008, Bonferroni) (F_3,32_ = 4.72, P = 0.008, one-way ANOVA), these elevations persisted with tirzepatide treatment (P = 0.128, Bonferroni). However, the dopamine levels in the tirzepatide-cocaine treated mice were not statistically different from vehicle controls (P = 0.804, Bonferroni). In the LS, cocaine produced substantial dopamine increases (P < 0.001, Bonferroni) (F_3,32_ = 17.11, P < 0.001, one-way ANOVA), which tirzepatide appeared to normalise (P < 0.001, Bonferroni) towards control concentrations (P = 0.149, Bonferroni).

GABA concentrations displayed cocaine-induced suppression across multiple regions that tirzepatide tended to reverse (Fig. 4B). Within the NAc, cocaine reduced GABA levels (P = 0.002, Bonferroni) (F_3,32_ = 6.32, P = 0.002, one-way ANOVA), reductions tirzepatide treatment mitigated (P = 0.023, Bonferroni) towards vehicle concentrations (P > 0.999, Bonferroni). Similar suppression occurred in the VTA (P = 0.013, Bonferroni) (F_3,32_ = 4.41, P = 0.011, one-way ANOVA), where tirzepatide again appeared to reverse the effect (P = 0.039, Bonferroni), bringing levels closer to vehicle control levels (P > 0.999, Bonferroni). The PFC also showed cocaine-induced GABA alterations (P = 0.007, Bonferroni) (F_3,32_ = 4.93, P = 0.006, one-way ANOVA), though tirzepatide's influence here did not reach significance (P = 0.099, Bonferroni). The LS mirrored the NAc and VTA pattern, with cocaine decreasing GABA concentrations (P = 0.007, Bonferroni) (F_3,32_ = 5.04, P = 0.006, one-way ANOVA) and tirzepatide counteracting this suppression (P = 0.020, Bonferroni) towards vehicle control levels (P > 0.999, Bonferroni).

Glutamate displayed more limited regional responsiveness to tirzepatide (Fig. 4C). Cocaine elevated glutamate across three regions: NAc (P = 0.005, Bonferroni) (F_3,32_ = 5.38, P = 0.004, one-way ANOVA), VTA (P = 0.009, Bonferroni) (F_3,32_ = 4.96, P = 0.006, one-way ANOVA), and LS (P = 0.002, Bonferroni) (F_3,32_ = 6.22, P = 0.002, one-way ANOVA). Tirzepatide's effects on glutamate appeared more selective than its actions on dopamine or GABA. Only within the LS did tirzepatide significantly reduce glutamate (P = 0.022, Bonferroni) towards concentrations resembling vehicle (P > 0.999, Bonferroni). In the NAc the tirzepatide effect was not statistically different from the cocaine group (P = 0.098, Bonferroni), while VTA elevations persisted largely unchanged. No significant glutamate effects emerged in the PFC (F_3,32_ = 1.30, P = 0.292, one-way ANOVA).

To examine neurochemical interdependence with drug treatment, we performed PCA on the two cocaine-exposed groups. PC1 explained 44% of the variance and discriminated subjects by treatment (t_16_ = 4.64, P < 0.001, unpaired t-test; Fig. 4D). Factor loadings suggested that neurotransmitters in the VTA and LS correlated strongly with the underlying principal component, with these regions appearing to account for much of the observed variance (Fig. 4D). PC2 explained 21% of the variance but showed no differences in component loading between treatment groups. Correlation analysis using Pearson coefficients demonstrated interregional associations, with particularly strong connectivity between the VTA and LS (Fig. 4E). PCA incorporating all four treatment groups appears in Supplementary Figure S13. PCA incorporating locomotion in the cocaine-treated groups is shown in Supplementary Figure S14.

These *ex vivo* measurements suggest tirzepatide may modulate cocaine-induced neurochemical alterations across mesocorticolimbic circuits and the lateral septum, with the VTA and LS showing particularly pronounced changes. However, whether these tissue-level changes directly contribute to the behavioural effects observed in Exp 13 remains unclear, though the correspondence between neurochemical and behavioural findings is consistent with such a possibility.

## Discussion

Given the pressing need for effective treatments for CUD, our study demonstrates that tirzepatide dose-dependently attenuates behaviours associated with cocaine's stimulatory dopaminergic effects across cocaine doses with different reinforcing efficacy (10 and 20 mg/kg). These effects appear dissociable from general behavioural suppression or malaise, as tirzepatide did not suppress spontaneous locomotion or induce pica responses, a rodent measure of nausea. The 30 nmol/kg dose did however reduce food intake and body weight, indicating additional effects on ingestive behaviour. These behavioural changes were accompanied by normalisation of cocaine-induced neurochemical alterations in circuits implicated in addiction-related behaviours. PCA analysis suggested the VTA and LS contribute to variance between cocaine-exposed groups, though more thorough circuit-level interpretations require further investigation.

Tirzepatide dose-dependently attenuated acute locomotor responses to cocaine (10 mg/kg). While the 3 nmol/kg dose was unable to significantly decrease locomotor stimulation (Exp 1), both 30 and 70 nmol/kg doses reduced the stimulatory effect of cocaine (Exp 2–3). Effect sizes were comparable to those recently reported for the GLP-1R agonist semaglutide.^26^ To explore whether this attenuation persisted against a higher cocaine challenge, we tested tirzepatide (30 nmol/kg) with a higher cocaine dose (20 mg/kg; Exp 4). Cocaine-induced locomotor stimulation remained attenuated even at this higher dose, indicating the effect extends beyond lower cocaine doses. Since both GLP-1R^58^^,^^59^ and GIPR^60^^,^^61^ are expressed within mesocorticolimbic circuits, we asked whether this dopamine-related^56^^,^^57^ behavioural attenuation extended beyond locomotion. Using CPP, tirzepatide (30 nmol/kg) decreased the expression of place preference for both the 10 mg/kg (Exp 5) and 20 mg/kg (Exp 6) cocaine doses. Tirzepatide thus attenuated dopamine-related behaviours across two distinct paradigms and different cocaine doses. This aligns with prior rodent studies showing that GLP-1R agonists reduce cocaine-related responses,^18^, ^19^, ^20^, ^21^, ^22^, ^23^, ^24^, ^25^, ^26^, ^27^ possibly through modulation of dopamine pathways.^18^^,^^21^, ^22^, ^23^^,^^25^^,^^26^^,^^62^ To test this hypothesis, we measured changes in cocaine-evoked dopamine levels in the NAc shell using *in vivo* microdialysis. Cocaine robustly elevated NAc shell dopamine with both doses (10 mg/kg; Exp 7 and 20 mg/kg; Exp 8). Tirzepatide pretreatment attenuated these elevations significantly, supporting an inhibitory modulatory effect on mesocorticolimbic dopamine signalling. Our recent findings extend this: tirzepatide also reduced alcohol-induced dopamine release in the NAc shell,^34^ regardless of whether alcohol was administered systemically or locally in NAc shell via reverse microdialysis, indicating that dopamine modulation may be a shared mechanism across different addictive drugs. To assess whether these findings translated to self-administration paradigms, we examined fixed ratio and progressive ratio responding in rats. Tirzepatide dose-dependently reduced cocaine self-administration (3 and 30 nmol/kg; Exp 9), indicating possible interference with mesocorticolimbic processes that drive drug-taking behaviour.^6^^,^^9^^,^^63^, ^64^, ^65^ Progressive ratio testing further showed that tirzepatide (30 nmol/kg) lowered motivation to self-administer cocaine, a behaviour closely linked to dopamine signalling.^6^^,^^56^^,^^57^ Our *ex vivo* tissue analysis complemented these *in vivo* findings: dopamine levels were normalised in the NAc, VTA and LS of tirzepatide-treated mice (30 nmol/kg) following the locomotor sensitisation to cocaine (10 mg/kg; Exp 13). Collectively, these findings spanning suppression of dopamine-related behaviours and attenuated cocaine-evoked dopamine levels, *in vivo* and *ex vivo,* suggest tirzepatide may exert a modulatory influence on mesolimbic dopamine circuitry as a potential underlying mechanism. The persistence of tirzepatide's attenuating effects even at higher cocaine doses suggests it may alter cocaine's reinforcing efficacy, aligning with prior work on GLP-1R agonists showing downward shifts in cocaine's dose–response curve.^27^ We acknowledge, however, that additional cocaine doses would be needed in future studies to establish this effect more thoroughly.

Beyond these acute effects on cocaine's dopamine-related responses, relapse prevention represents a critical clinical challenge in addiction treatment.^1^^,^^2^^,^^8^^,^^11^ To examine whether tirzepatide might also reduce relapse risk, we next investigated its effects on reinstatement of cocaine seeking. While suppressing cocaine's reinforcing effects is therapeutically interesting, our findings on relapse-related behaviours may hold greater clinical promise. Tirzepatide suppressed drug-primed reinstatement in both the self-administration model (rats; Exp 11) and CPP model (mice; Exp 12). This effect also extends beyond cocaine: our recent work shows tirzepatide attenuates relapse-like alcohol drinking and cue-induced place preference for alcohol following forced abstinence,^34^ suggesting possible effects on both drug memory and cue reactivity.^3^, ^4^, ^5^^,^^66^ The breadth and cross-species consistency of these findings point to relapse prevention as a particularly promising translational avenue, although it should be acknowledged that animal models cannot fully recapitulate human relapse. Future work should aim to further explore this clinical potential.

Although our relapse-related findings are encouraging, repeated cocaine exposure also induces neuroadaptive changes that persist beyond acute stimulatory effects.^6^^,^^10^ Whether tirzepatide could modulate more long-term neurochemical alterations remained unexplored. To address this, we employed the locomotor sensitisation model and analysed tissue neurotransmitter levels from the same experiment (Exp 13) to get a rough estimation of potential effects of tirzepatide on cocaine-altered neurochemistry. Tirzepatide blocked the expression of cocaine-induced locomotor sensitisation, extending its effects beyond acute drug-evoked behaviours to potentially encompass neuroadaptive processes. Our *ex vivo* tissue analysis revealed that tirzepatide normalised cocaine-evoked alterations in dopamine, GABA and glutamate across multiple regions. However, these data warrant cautious interpretation: *ex vivo* tissue levels reflect both intracellular and extracellular neurotransmitter pools,^67^^,^^68^ and thus do not directly correlate with functional synaptic transmission or receptor engagement. Whether tirzepatide's normalisation of *post-mortem* levels of neurotransmitters represents actual *in vivo* temporal dynamics of tirzepatide's effects remains uncertain. Notwithstanding this limitation, tirzepatide potential modulation cocaine-induced GABA and glutamate signalling is interesting as these systems are implicated CUD pathophysiology.^2^^,^^63^ GLP-1R agonists have also been shown to influence inhibitory GABA tone and excitatory glutamate signalling within several brain circuits,^20^^,^^24^^,^^25^^,^^35^^,^^69^, ^70^, ^71^, ^72^, ^73^, ^74^, ^75^ including studies with the cocaine,^20^^,^^24^^,^^25^ potentially explaining the neurochemical data. PCA indicated that neurochemical changes in the VTA and LS explained the variance between treatment groups, raising the possibility that these regions interact to mediate part of tirzepatide's effects. Whether tirzepatide directly targets mesolimbic circuits remains unclear. The limited blood–brain barrier (BBB) penetration of long-acting incretin agonists^76^^,^^77^ suggests indirect mechanisms are more likely at play. This constraint points toward regions where BBB integrity is compromised, specifically areas proximal to circumventricular organs (CVOs), as probable sites of action. The LS is a plausible candidate: it is adjacent to the subfornical organ (a CVO),^78^, ^79^, ^80^ contains both GLP-1R^58^^,^^59^ and GIPR,^60^^,^^61^ and maintains connections to the NAc and VTA.^53^^,^^81^, ^82^, ^83^, ^84^, ^85^, ^86^, ^87^ Preclinical work implicates the LS in GLP-1R-mediated responses to addictive drugs, including cocaine.^20^^,^^35^^,^^62^^,^^78^^,^^88^ These studies further suggest a modulatory role in mesolimbic dopamine signalling, possibly mediated by GABAergic mechanisms.^35^ GLP-1R agonist exenatide also reduced septal cue reactivity in patients with alcohol use disorder,^88^ hinting at possible clinical relevance. Whether the LS serves as a site of action for tirzepatide, however, remains to be established. Other CVO-proximal regions, such as the dorsal vagal complex, and the hypothalamus, may similarly contribute.^76^^,^^77^^,^^89^

Several methodological considerations warrant explicit acknowledgement. Our exclusive use of male animals limits generalisability of findings. Sex differences in addiction have been reported,^36^^,^^37^^,^^42^ though whether tirzepatide's effects on cocaine-related responses transfer across sexes remains uncertain. Gonadal hormones, particularly oestrogen, modulate both GLP-1R function^39^^,^^41^^,^^90^ and cocaine responses,^36^^,^^37^^,^^42^ and sex-dependent effects related to GLP-1 in alcohol research have been observed.^34^^,^^35^^,^^40^^,^^91^^,^^92^ Whether this extends to GIPR, or whether tirzepatide's efficacy varies with the oestrous cycle, for example, is unknown. Future studies should include female subjects to establish whether these findings translate across sexes. Additional cocaine doses would also strengthen confidence in tirzepatide's effects on reinforcing efficacy, as we cannot exclude dose-dependent breakthrough at higher doses. Another limitation of our study is the unexplored relative contributions of GIPR versus GLP-1R agonism to tirzepatide's effects. Whether tirzepatide's efficacy arises from GLP-1R agonism, GIPR agonism, or synergistic interaction between both receptors cannot be determined from the current data. However, several complexities complicate straightforward investigation. First, the available pharmacological tools do not permit a clean mechanistic dissection. Tirzepatide is a biased GLP-1R agonist that preferentially activates cAMP signalling,^32^ whereas approved GLP-1R monoagonists engage both cAMP and β-arrestin pathways,^32^^,^^33^^,^^93^^,^^94^ thereby activating distinct downstream signalling cascades. Consequently, neither a mono GLP-1R agonist, a GIPR agonist, nor their combination can recapitulate tirzepatide's unimolecular pharmacology. Moreover, establishing dose equivalence across these structurally and pharmacodynamically distinct compounds is itself problematic. Second, both GIPR agonism and antagonism in combination with GLP-1R agonism paradoxically decrease food intake and body weight,^95^ complicating interpretation of GIPR's role within a dual-agonist context. Third, potential differences in pharmacokinetics and receptor internalisation between long-acting incretin agonists and their respective receptor antagonists may make effective pharmacological blockade difficult. For example, semaglutide's effects on alcohol intake were not blocked by direct GLP-1R antagonism.^96^ Fourth, either biased GLP-1R agonism or GIPR agonism in combination with unbiased GLP-1R agonism appears to enhance tolerability in metabolic contexts.^50^^,^^93^^,^^94^^,^^97^ This raises a fundamental question: whether isolating individual receptors would obscure rather than illuminate a clinically relevant synergistic mechanism. Thus, future studies need careful consideration to untangle this complexity. Tirzepatide also reduced food intake at the 30 nmol/kg dose. However, consistent with prior work^34^ tirzepatide did not affect spontaneous locomotion or exploratory behaviours, and kaolin consumption remained unchanged, arguing against malaise or generalised behavioural suppression. We additionally observed substantial heterogeneity in individual responses: some animals showed robust cocaine suppression following tirzepatide, while others showed minimal effect. This variability did not correlate with any certain baseline characteristics, though future studies could investigate possible factors predicting treatment response, a question with clinical relevance given that incretin-based therapeutics may only benefit certain subpopulations of individuals with CUD. Despite these limitations, convergent effects across multiple behavioural paradigms and species provide preliminary evidence for tirzepatide's broad action on cocaine-related responses. The clinical landscape for long-acting incretin mimetics continues to expand. Semaglutide reduces alcohol consumption across species: from rodents^75^^,^^91^ through non-human primates^98^ to humans,^99^^,^^100^ offering initial but promising evidence for broader therapeutic utility in substance use disorders. Although tirzepatide research in addiction remains early-stage,^34^^,^^101^^,^^102^ targeting multiple pathways simultaneously may offer additional therapeutic advantages that warrants continued exploration.

In conclusion, our findings indicate that tirzepatide reduces multiple dimensions of cocaine use: voluntary drug taking, reward processing, motivation, and drug seeking, while normalising neurochemical alterations across several neurotransmitter systems in key mesolimbic circuits. Importantly, our data suggest tirzepatide may also impact neuroadaptive changes that occur from repeated cocaine use which are implicated in drug craving and relapse vulnerability. These effects, observed across species and behavioural paradigms, coupled with tirzepatide's established clinical approval, position this incretin polyagonist as a promising candidate for CUD that merits further investigation.

## Contributors

**Christian E Edvardsson:** Conceptualisation, Methodology, Investigation, Formal analysis, Data curation, Writing—original draft, Writing—review & editing, Visualisation, Project administration, Data verification. **Xinming Zhang:** Investigation, Formal analysis, Data curation, Writing—review & editing, Data verification. **Thaynnam A Emous:** Investigation, Formal analysis, Data curation, Writing—review & editing, Data verification. **Louise Adermark:** Investigation, Formal analysis, Writing—review & editing, Data verification. **Sarah Witley:** Investigation, Writing—review & editing. **Mia Ericson:** Writing—review & editing, Resources, Methodology. **Heath D Schmidt:** Conceptualisation, Formal analysis, Data curation, Methodology, Writing—review & editing, Resources, Funding acquisition, Data verification. **Elisabet Jerlhag:** Conceptualisation, Formal analysis, Data curation, Writing—review & editing, Resources, Funding acquisition, Data verification.

All authors have read and approved the final version of the manuscript.

## Data sharing statement

Data collected for the study will be made available and shared with others though contact at the following address: elisabet.jerlhag@pharm.gu.se. The data will be shared with researchers who want to do additional analysis of the data and therefore the data will be shared after approval of a proposal, and with a signed data access agreement.

## Declaration of interests

Authors declare that they have no competing interests.
