# Supplementary material for: Tirzepatide attenuates mesolimbic cocaine-evoked dopamine levels and reduces cocaine taking, motivation and seeking behaviours in male rodents
Source: eBioMedicine. 2026 Mar 23;126:106219. doi: 10.1016/j.ebiom.2026.106219 (PMC13049583; doi:10.1016/j.ebiom.2026.106219)
Supplement: Supplementary Information [file mmc1.doc]

**SUPPLEMENTARY INFORMATION**

**METHODS**

**Open field, elevated plus maze and light-dark box tests in mice**

To assess whether tirzepatide in itself affects general motor behaviours and anxiety-like responses in cocaine-naïve mice (n=36, 12 per group), we utilised three complementary paradigms (open field, elevated plus maze (EPM) and the light-dark box test) with one week between each test using a between-subjects design, where each mouse received the same treatment for all tests. Following 60-minute habituation to the test room, mice were pretreated with vehicle or tirzepatide (30 or 70 nmol/kg) and then tested 30 minutes later.

Open field testing utilised the same apparatus as locomotor activity experiments but with increased lighting (50 lx). Mice were placed in a corner and allowed to freely explore the arena for 30 minutes. The EPM consisted of two open arms (5×30 cm) and two enclosed arms with light-blocking walls (20 cm high), elevated 40 cm above floor level and lit at 40 lx. Mice were placed in the centre and then allowed to investigate the arena for 5 minutes. The light-dark box test consisted of a two-chamber arena (50×25×25 cm) with a brightly lit compartment (250 lx) and an enclosed dark compartment (0 lx), separated by a wall with a central opening (7×7 cm). Mice were placed in the dark compartment at the start of the test and then left to freely explore the arena for 5 minutes. EPM and light-dark box tests were analysed utilising Observer XT software (Version 15, Noldus, Wageningen, Netherlands).

**Microdialysis surgery**

Mice were anaesthetised with isoflurane (Baxter, Apoteket AB, Gothenburg, Sweden), placed in a stereotaxic frame, and maintained on a heating pad. Local anaesthesia (Xylocaine with adrenaline, 10 mg/ml, 5 μg/ml; Pfizer Inc, Apoteket AB, Gothenburg, Sweden) was applied at the incision site. Carprofen (Rimadyl®, 5 mg/kg, AstraZeneca, Apoteket AB, Gothenburg, Sweden), 0.9% NaCl, and Viscotears were administered for pain management, rehydration, and eye protection. After exposing the skull, holes were drilled for the probe and anchoring screws. The probe was secured with dental cement (DENTALON® Plus, Agntho's AB, Lidingö, Sweden).

**Neurochemical tissue analysis of monoamines and amino acids**

Dissected tissue samples were homogenised in 0.1 M perchloric acid with 5.4 mM EDTA using an ultrasonicator (Sonifier Cell Disruptor B30, Branson Sonic Power Co., Danbury, CT, USA), then centrifuged (10,000 rpm, 5°C, 10 min) to obtain tissue-free supernatant containing neurotransmitters of interest.

*Monoamine analysis:* Supernatant aliquots were analysed for noradrenaline (NA), dopamine (DA), serotonin (5-HT), and their metabolites normetanephrine (NM), 3-methoxytyramine (3-MT), 3,4-dihydroxyphenylacetic acid (DOPAC), 5-hydroxyindoleacetic acid (5-HIAA), and homovanillic acid (HVA) using HPLC with electrochemical detection. In brief, the analytical method for monoamines utilised two isocratic chromatographic separations dedicated to amines or acids. The amines (NA, DA, NM, 3-MT, and 5-HT) were separated using two-dimensional reverse-phase ion-pair HPLC on two fully porous silica C18 reverse-phase columns (Luna C18(2), 20×2 mm and 50×2 mm, 3 µm particle size, 100 Å pore size, Phenomenex, Værløse, Denmark). The two mobile phases consisted of 86% (v/v) of a buffered aqueous solution (5 mM citric acid monohydrate, 10 mM di-sodium citrate trihydrate, 0.1 mM Na₂-EDTA, with 0.7 mM sodium-1-dodecanesulfonate additionally added to the second mobile phase, pH 5.5), 10% (v/v) acetonitrile, and 4% (v/v) tetrahydrofuran, delivered at flow rates of 0.300 ml/min and applied potential for the detector was 400 mV. The acids (DOPAC, 5-HIAA, and HVA) were separated using reverse-phase ion-pair HPLC on the same type of column (Luna C18(2), 50×2 mm). The mobile phase consisted of 92% (v/v) of a buffered aqueous solution (53.3 mM citric acid monohydrate, 13.2 mM di-potassium hydrogen phosphate trihydrate, 0.1 mM Na₂-EDTA, 0.2 mM sodium-1-dodecanesulfonate, pH adjusted to 2.95 with 5 M NaOH), and 8% (v/v) methanol, delivered at a flow rate of 0.300 ml/min and an applied potential of 600 mV.

*Amino acid analysis:* Amino acids (glutamate, γ-aminobutyric acid (GABA), glycine, taurine, serine and glutamine) were detected using a separate HPLC system with fluorescent detection. To avoid degradation, supernatant samples were here diluted 1:100 in a solution consisting of 20 mM sodium azide and ddH₂O before the analysis, which used a gradient HPLC system (UltiMate 3000, Thermo Fisher Scientific, Gothenburg, Sweden). Samples underwent preanalysis derivatisation with o-phthaldialdehyde/2-mercaptoethanol for 10 seconds, followed by separation on a 5 μm C18 Kinetex® column (4.6×150 mm, Phenomenex, Værløse, Denmark) with SecurityGard precolumn (Phenomenex). The gradient mobile phase consisted of: (A) 0.1 M sodium phosphate with 0.1 mM EDTA (pH 6.38, adjusted with phosphoric acid), (B) methanol (100%), (C) acetonitrile (50%), and (D) methanol (4%) with acetonitrile (1%), delivered at 2.5 ml/min flow rate. Column temperature was maintained at 39°C, with fluorescence detection at 348 nm excitation and 450 nm emission wavelengths.

Instrument control and data acquisition, for all three HPLC systems, were carried out using the Chromeleon™ Chromatography Data System (CDS) Software (Version 6, ThermoFisher Scientific, Gothenburg, Sweden).

**RESULTS**

**Tirzepatide does not alter exploration or cause anxiety-like behaviours in mice**

The open field test revealed no differences in distance travelled (F_2,33_=0.11, P=0.899, one-way ANOVA), vertical counts (F_2,33_=0.13, P=0.878, one-way ANOVA), time in centre zone (F_2,33_=0.06, P=0.942, one-way ANOVA), or centre zone entries (F_2,33_=0.50, P=0.612, one-way ANOVA) (**Supplementary Figure 1A-D**). We further observed no significant effects in the EPM test; open arm time (F_2,33_=2.49, P=0.099, one-way ANOVA) or open arm entries (F_2,33_=1.96, P=0.157, one-way ANOVA) (**Supplementary Figure 1E-F**). The light-dark box test further confirmed no effects *per se* on light compartment time (F_2,33_=0.77, P=0.471, one-way ANOVA) or light side entries (F_2,33_=0.31, P=0.737, one-way ANOVA) (**Supplementary Figure 1G-H**). These findings support the interpretation that tirzepatide does not cause its effect on cocaine-related responses by affecting spontaneous locomotion, exploration or inducing anxiety-like behaviours.

**Effect of tirzepatide on in vivo cocaine-evoked accumbal monoamine levels in mice**

In addition to the dopamine findings (Exp 7), tirzepatide (30 nmol/kg) similarly reduced cocaine (10 mg/kg)-induced increases in accumbal noradrenaline (treatment F_1,14_=7.77, P=0.015; time F_13,182_=54.32, P<0.001; interaction treatment × time F_13,182_=4.37, P<0.001, repeated measures two-way ANOVA followed by Bonferroni post hoc test; **Supplementary Figure 4A**) and serotonin (treatment F_1,14_=7.34, P=0.017; time F_13,182_=46.68, P<0.001; interaction treatment × time F_13,182_=2.39, P=0.006, repeated measures two-way ANOVA followed by Bonferroni post hoc test; **Supplementary Figure 4B**). Moreover, tirzepatide also lowered cocaine-elevated increases in 3-MT levels (treatment F_1,14_=12.01, P=0.004; time F_13,182_=30.34, P<0.001; interaction treatment × time F_13,182_=5.74, P<0.001, repeated measures two-way ANOVA followed by Bonferroni post hoc test; **Supplementary Figure 4C**), while NM, 5-HIAA, DOPAC, and HVA remained unchanged across treatments (repeated measures two-way ANOVA; **Supplementary Figure 4D-G**). AUC analyses (unpaired t-test) for all monoamines in Exp 7 are summarised in **Supplementary Figure 4H**. In Exp 8, tirzepatide (30 nmol/kg) did not statistically reduce cocaine (20 mg/kg)-induced increases in accumbal noradrenaline, serotonin, 3-MT, NM, 5-HIAA, DOPAC, or HVA (repeated measures two-way ANOVA; **Supplementary Figure 5A-G**). AUC analyses (unpaired t-test) for all monoamines in Exp 8 are summarised in **Supplementary Figure 5H**.

**Effect of tirzepatide on ex vivo neurochemistry in additional brain regions and neurotransmitters in cocaine-sensitised mice**

Additional brain regions in Exp 13 revealed selective tirzepatide (30 nmol/kg) effects on cocaine (10 mg/kg)-induced neurochemical changes. The dorsal lateral striatum (DLS) showed cocaine-induced dopamine increases (P=0.005, Bonferroni) (F_3,32_=5.35, P=0.004, one-way ANOVA) that tirzepatide reversed (P=0.048, Bonferroni), restoring levels to vehicle ranges (P>0.999, Bonferroni). In contrast, the amygdala displayed cocaine effects on dopamine (P=0.027, Bonferroni) (F_3,32_=3.62, P=0.023, one-way ANOVA) that persisted without tirzepatide modification (**Supplementary Figure 8A**). Glycine concentrations showed region-specific effects (**Supplementary Figure 9A**). Cocaine increased glycine in both nucleus accumbens (NAc) (P=0.023, Bonferroni) (F_3,32_=3.61, P=0.024, one-way ANOVA) and lateral septum (LS; P=0.010, Bonferroni) (F_3,32_=4.65, P=0.008, one-way ANOVA). Tirzepatide significantly reduced these elevations in the LS (P=0.022, Bonferroni), normalising concentrations to control levels (P>0.999, Bonferroni), while NAc alterations remained unaffected (P=0.729, Bonferroni). Noradrenaline concentrations increased with cocaine specifically in the prefrontal cortex (PFC; P=0.003, Bonferroni) (F_3,32_=5.83, P=0.003, one-way ANOVA), though tirzepatide had no significant effect on this response (**Supplementary Figure 9B**). Other brain regions and neurotransmitters showed minimal treatment effects (**Supplementary Figures 8-12**).

Principal component analysis (PCA) across all four groups examined neurochemical interdependence with drug exposure. PC1 explained 45% of total variance, while PC2 explained 12% (**Supplementary Figure 13A-B**). PC1 discriminated subjects by treatment (F_3,32_=36.60, P<0.001, one-way ANOVA), with significant differences in component loading between groups (Veh-Veh vs. Tzp-Veh, P>0.999; Veh-Veh vs. Veh-Coc, P<0.001; Veh-Coc vs. Tzp-Coc, P<0.001; Tzp-Veh vs. Tzp-Coc, P=0.052; Veh-Veh vs. Tzp-Coc, P=0.046, Bonferroni post hoc test; **Supplementary Figure 13C**). PC2 showed no treatment group differences (F_3,32_=0.10, P=0.957, one-way ANOVA; **Supplementary Figure 13C**). Factor loadings showed GABA presented a negative correlation with PC1, while dopamine in the ventral tegmental area (VTA; 81%) and LS (82%) contributed strongly to the variance (**Supplementary Figure 13D**). PC2 accounted for 12% of variance, with NAc glutamate as the main contributor (67%) (**Supplementary Figure 13E**). Correlation analysis using Pearson's correlation coefficient revealed strong associations between neurotransmitters both within and between brain regions. VTA dopamine correlated significantly with all analysed neurotransmitters and brain regions (**Supplementary Figure 13F**).

In an attempt to identify neurochemical associations to the locomotor stimulatory properties of cocaine, a sub analysis was performed comparing tissue analysis with the distance travelled on the challenge day (day 11) for the same individuals. Indeed, while tirzepatide reduced cocaine-induced locomotion, interindividual variability was observed (**Supplementary Figure 14A**). For this analysis, the DLS, amygdala and DMS were included based on their acknowledged involvement in behavioural sensitisation to psychostimulants, together with the VTA, NAc, PFC and LS.

PCA demonstrated that factor loading for locomotor activity completely overlapped with glutamate in LS and VTA, with dopamine going in the same direction (**Supplementary Figure 14B**). Factor loading for LS GABA went in the opposite direction (**Supplementary Figure 14B**).

Pearson’s correlation analysis confirmed a significant correlation between total distance travelled and neurochemical analysis performed in the VTA for both dopamine (P=0.009) glutamate (P=0.021) and GABA (P=0.008) (**Supplementary Figure 14C**). However, the most striking correlation was found for the LS, where dopamine and glutamate demonstrated a positive association (dopamine: P<0.001; glutamate: P<0.001), while GABA was inversely correlated with locomotion (P <0.001) (**Supplementary Figure 14C**). No significant association was found between locomotion and neurochemistry for the other brain regions assessed.

Importantly, correlation analysis performed for mice treated with vehicle demonstrated no association between distance travelled and neurochemistry in LS (**Supplementary Figure 14D**). In these mice, the distance travelled only correlated with glutamate in PFC (P=0.035) (**Supplementary Figure 14D**).

Since neurochemical analysis represents tissue levels and can not be directly translated into synaptic neurotransmission, and as tissue was collected after the locomotor behaviour, these findings should be interpreted cautiously. Still, the strong association between the LS and distance travelled still indicates that LS play a role in modulating locomotor responses to cocaine.

**SUPPLEMENTARY FIGURES**


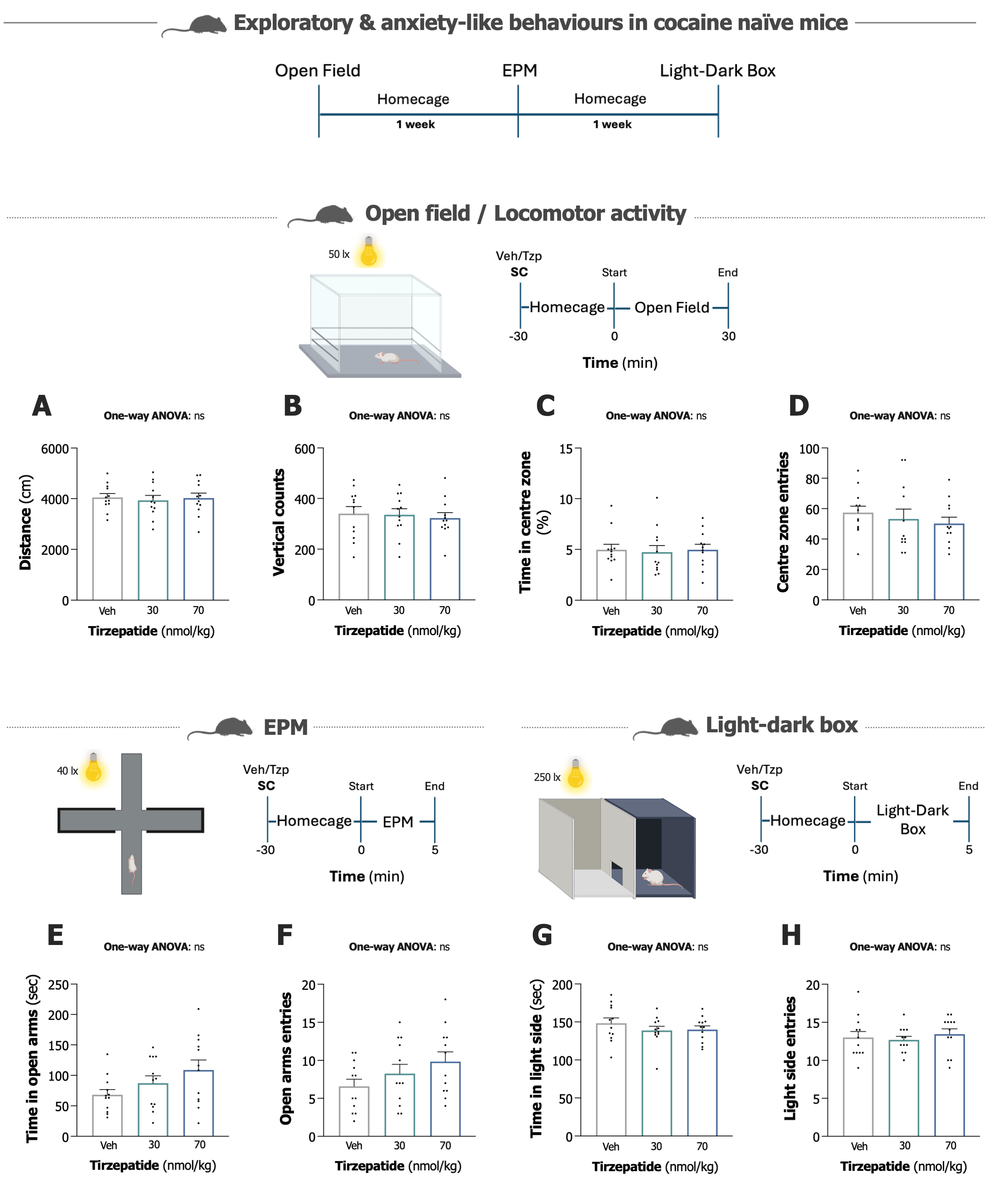
**Supplementary Figure 1**

**Supplementary Figure 1. Tirzepatide effects on exploration and anxiety-like behaviours in cocaine-naïve mice.**

**A-D.** No significant effects are noticed in the open field test on distance travelled, vertical counts, time spent in centre zone, or centre zone entries following tirzepatide administration (30 or 70 nmol/kg) compared to vehicle (n=12/group, one-way ANOVA). **E-F.** Tirzepatide treatment does not lead to any significant changes in time spent in open arms or open arm entries in the elevated plus maze (EPM) test (n=12/group, one-way ANOVA). **G-H.** Neither does tirzepatide affect any parameters in the light-dark box test (n=12/group, one-way ANOVA). Data are presented as mean±SEM with individual data points shown where appropriate. ns; non-significant.

**Supplementary Figure 2**

**
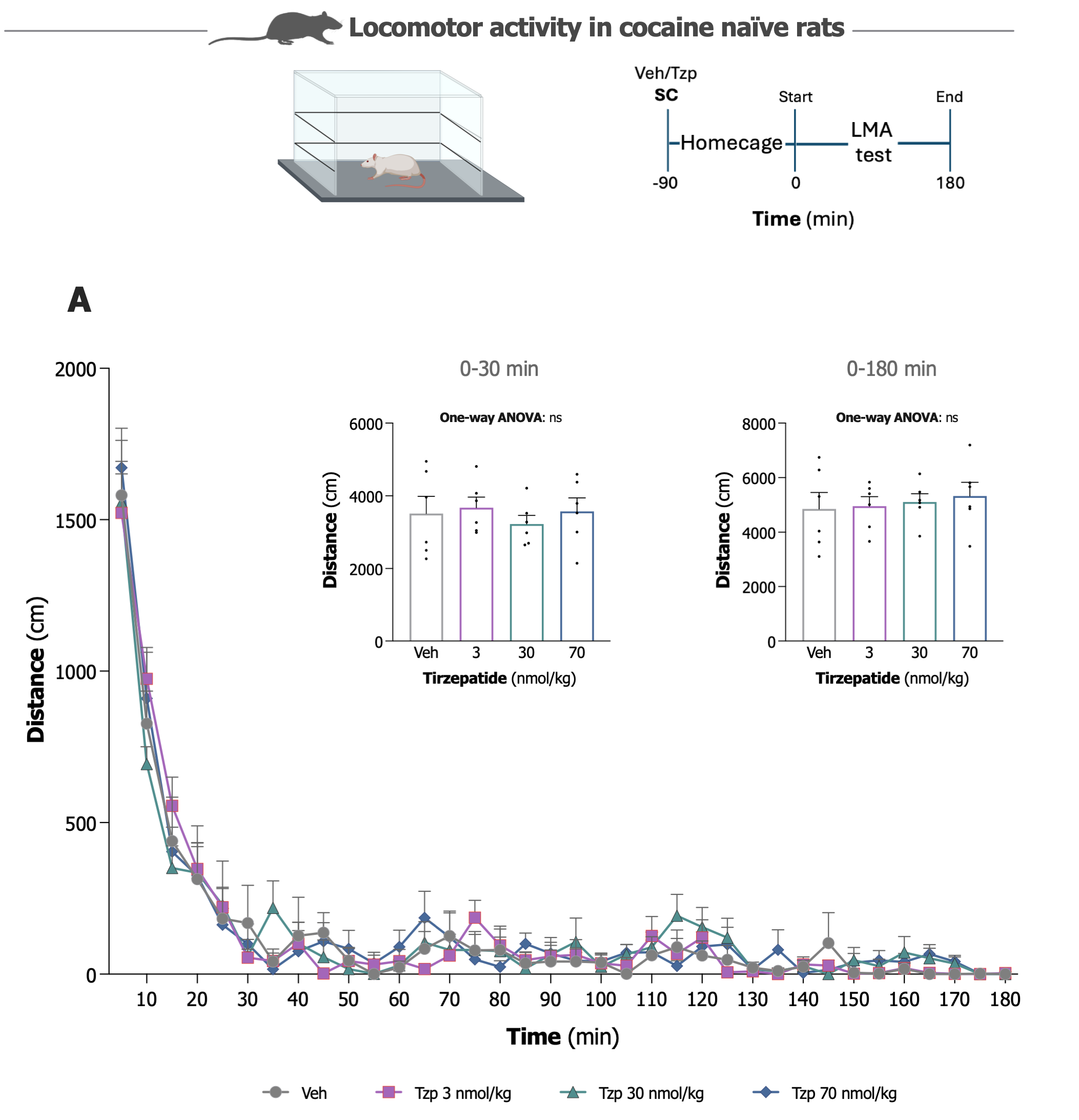
**

**Supplementary Figure 2. Tirzepatide’s effects on spontaneous locomotor activity in cocaine-naïve rats.**

**A.** Pretreatment with tirzepatide (Tzp; 3, 30 or 70 nmol/kg) or vehicle 90 min before testing in the locomotor activity test over three hours did not significantly differ in distance travelled at any dose, either during the first 30 minutes or at the full 3-hour (180 min) mark (n=6/group, one-way ANOVA). Data show individual data points with mean±SEM. ns; non-significant.

**Supplementary Figure 3**

**
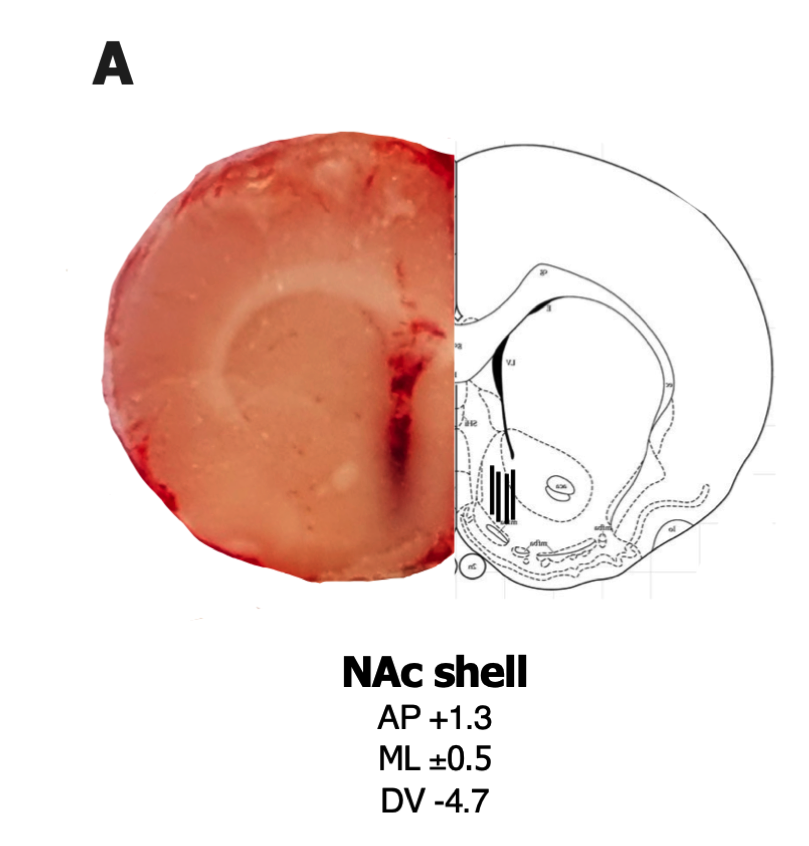
**

**Supplementary Figure 3. Probe placements.**

**A.** Schematic representation of probe placements in the NAc shell in mice, including stereotaxic coordinates.


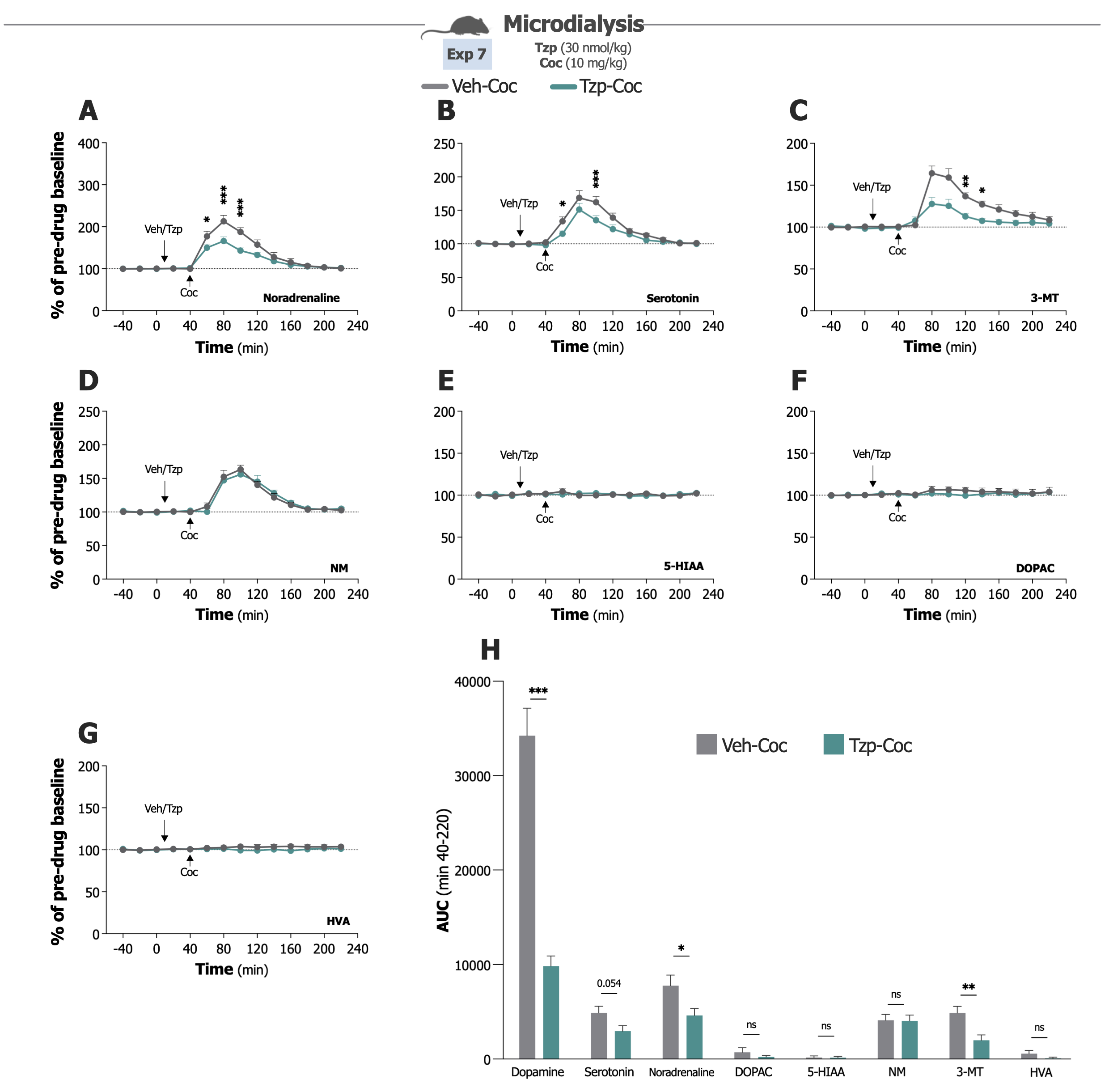
**Supplementary Figure 4**

**Supplementary Figure 4. Accumbal monoamine levels in mice following tirzepatide (30 nmol/kg) treatment and cocaine administration (10 mg/kg).**

Cocaine (Coc; 10 mg/kg) administration evokes significant increases in **A.** noradrenaline, **B.** serotonin, and **C.** 3-MT in the NAc shell, which tirzepatide (Tzp; 30 nmol/kg) treatment significantly attenuates compared to vehicle (n=8/group, repeated measures two-way ANOVA (time x treatment) followed by Bonferroni post hoc test). No significant effects between treatments occur with **D.** NM, **E.** 5-HIAA, **F.** DOPAC, and **G.** HVA (n=8/group, repeated measures two-way ANOVA (time x treatment). **H.** AUC analysis of all monoamines (n=8/group, unpaired t-test). Data are presented as mean ± SEM. ns; non-significant, *P<0.05, **P<0.01, ***P<0.001.


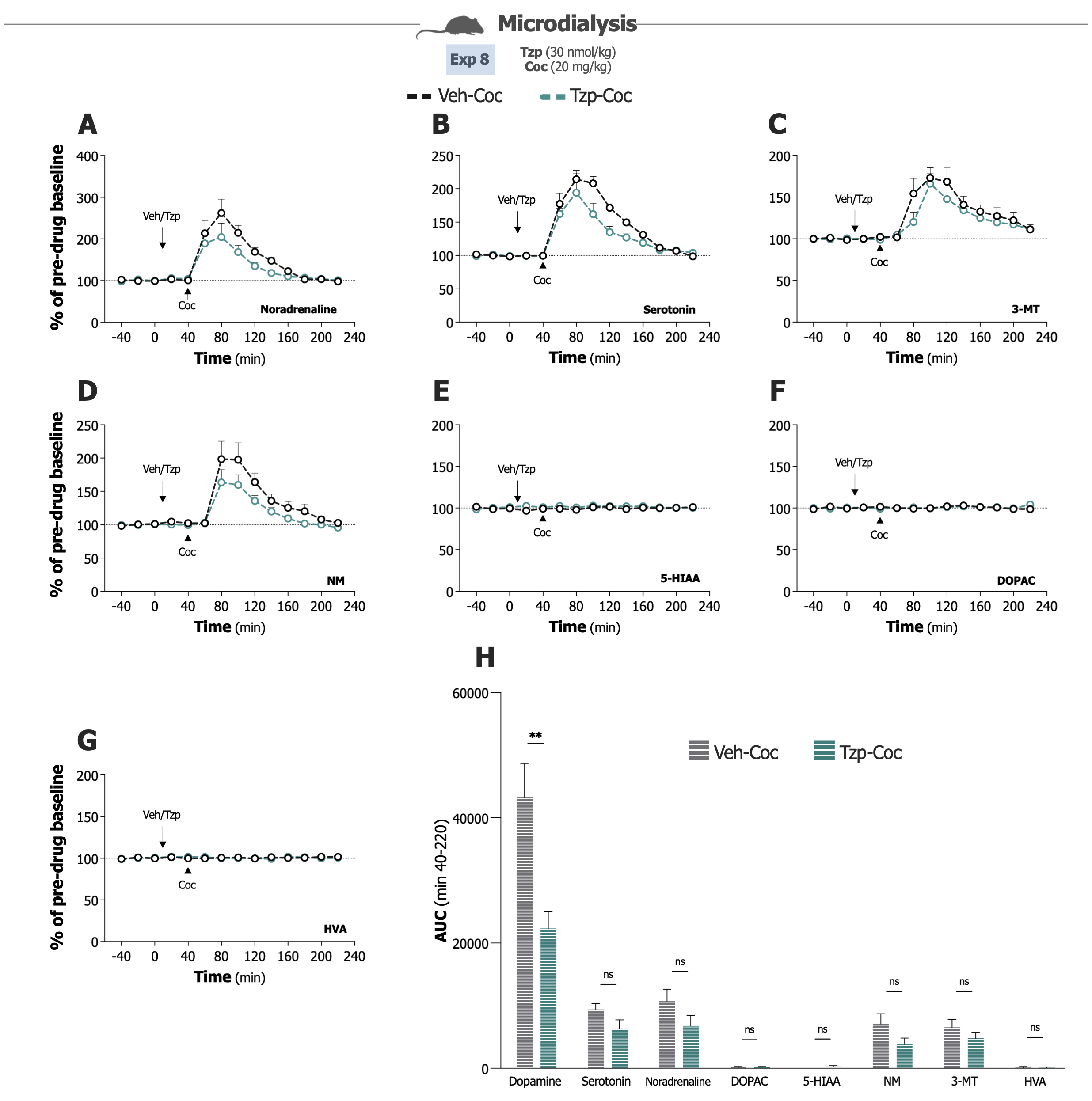
**Supplementary Figure 5**

**Supplementary Figure 5. Accumbal monoamine levels in mice following tirzepatide (30 nmol/kg) treatment and cocaine administration (20 mg/kg).**

No significant effects between tirzepatide (Tzp; 30 nmol/kg) and vehicle occur with **A.** noradrenaline, **B.** serotonin, and **C.** 3-MT, **D.** NM, **E.** 5-HIAA, **F.** DOPAC, and **G.** HVA on cocaine (Coc; 20 mg/kg)-evoked monoamine levels in the NAc shell (n=7/group, repeated measures two-way ANOVA (time x treatment)). **H.** AUC analysis of all monoamines (n=7/group, unpaired t-test). Data are presented as mean ± SEM. ns; non-significant, **P<0.01.

**Supplementary Figure 6**

**
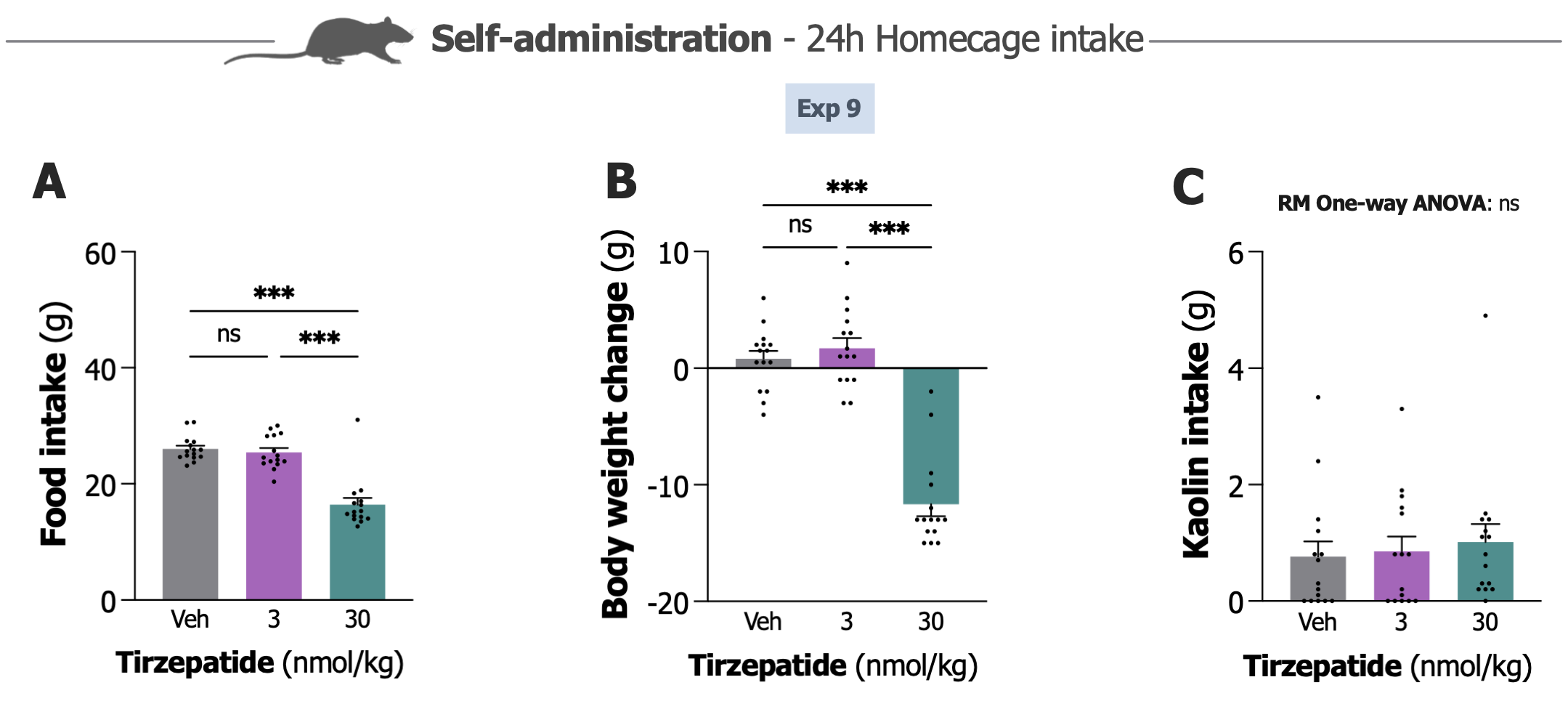
**

**Supplementary Figure 6. Tirzepatide effects on ingestive behaviour and body weight following cocaine self-administration tests in rats.**

**A-B.** The 30 nmol/kg dose of tirzepatide significantly reduces food intake as well as body weight 24 hours post-treatment compared to both vehicle and the 3 nmol/kg dose. The lower dose does not statistically differ from vehicle in either food intake or body weight change (n=15/group, repeated measures one-way ANOVA followed by Bonferroni post hoc test). **C.** Tirzepatide did not elicit pica (i.e., increase kaolin intake) in cocaine-experienced rats (n=15/group, repeated measures one-way ANOVA followed by Bonferroni post hoc test). Data are presented as mean±SEM. ns; non-significant, ***P<0.001.


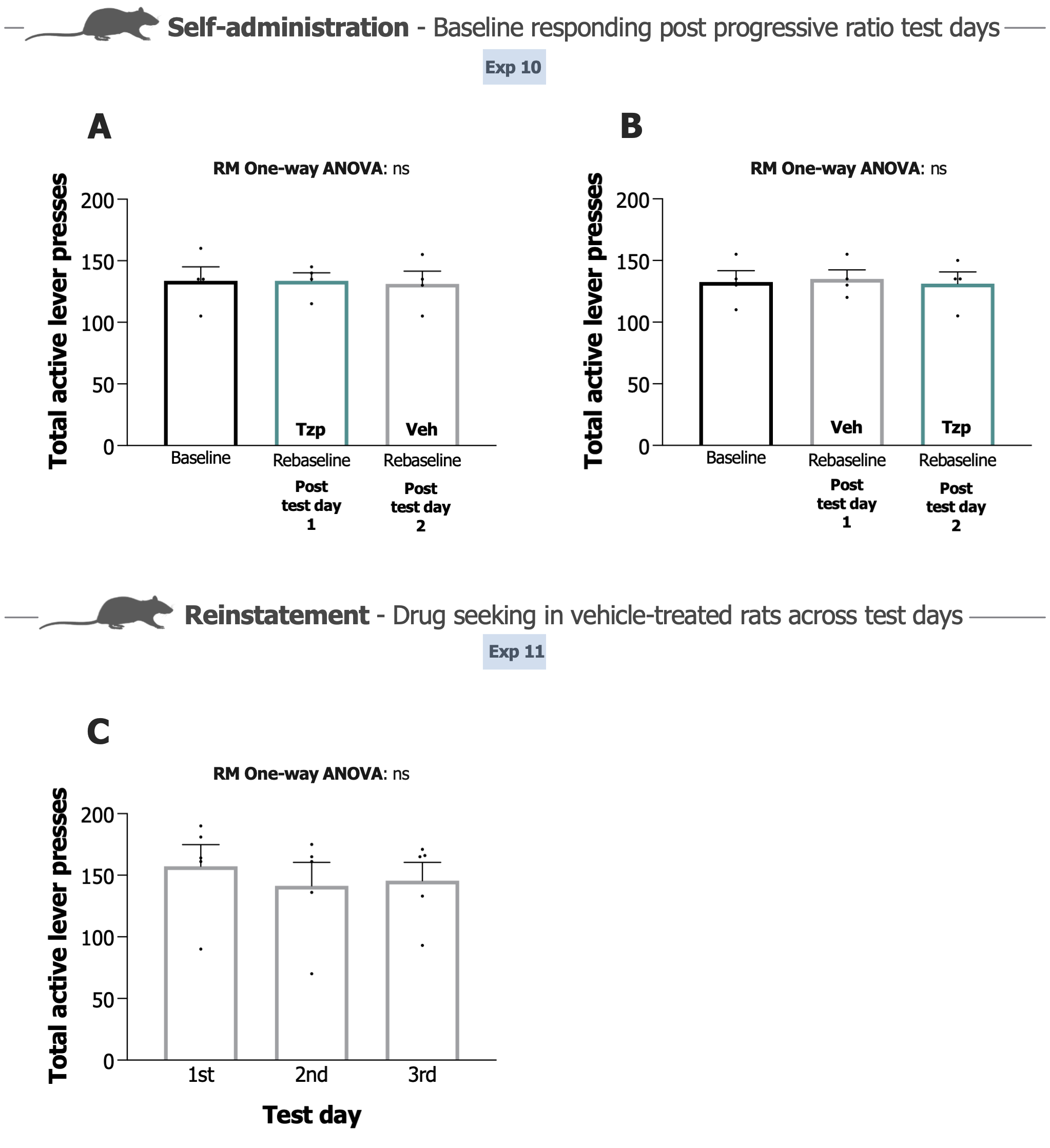
**Supplementary Figure 7**

**Supplementary Figure 7. Rebaseline data the day after the progressive ratio test days and drug-seeking behavior in vehicle-treated rats across test sessions.**

**A-B.** There were no effects of treatment on baseline FR5 responding following PR test sessions (n=4/group, repeated measures one-way ANOVA). **C.** There were no statistical differences in drug-seeking behavior between test days in the vehicle-treated rats (n=5/day, repeated measures one-way). Data are presented as mean±SEM. ns; non-significant.


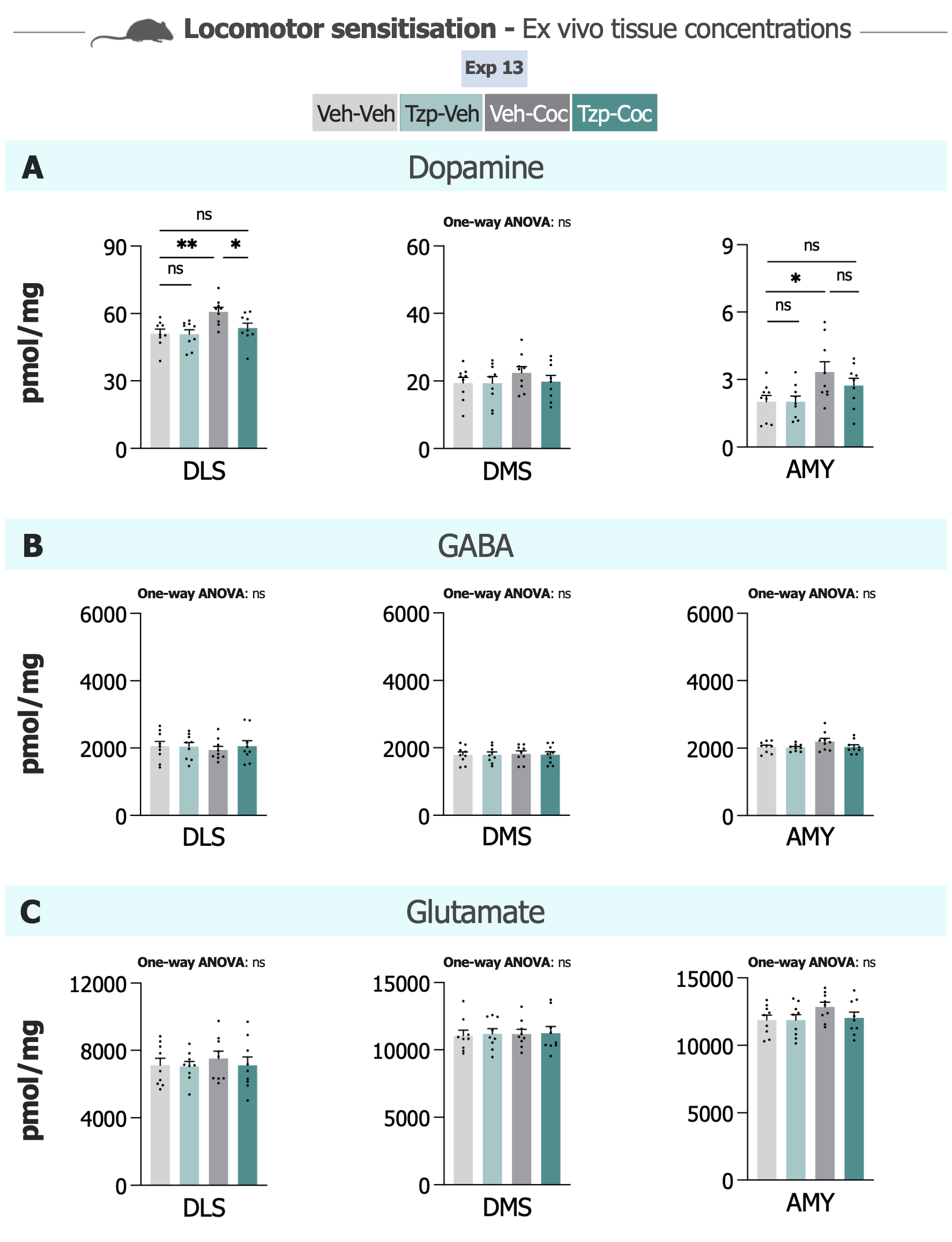
**Supplementary Figure 8**

**Supplementary Figure 8. Dopamine, GABA and glutamate alterations in additional brain regions following tirzepatide treatment in cocaine-sensitised mice.**

**A-C.** Ex vivo tissue concentration analysis of dopamine, GABA and glutamate in DLS, DMS and amygdala (AMY) following repeated cocaine (Coc; 10 mg/kg) or vehicle exposure with tirzepatide (Tzp; 30 nmol/kg) or vehicle treatment (n=9/group, one-way ANOVA followed by Bonferroni post hoc test). **A.** Dopamine concentrations reveal cocaine-induced elevations in the DLS and amygdala. Tirzepatide restores these increases in the DLS, while amygdala levels remain elevated. **B-C.** GABA and glutamate concentrations remain largely unaffected. Data are presented as mean±SEM. ns; non-significant, *P<0.05, **P<0.01.

**Supplementary Figure 9**

**
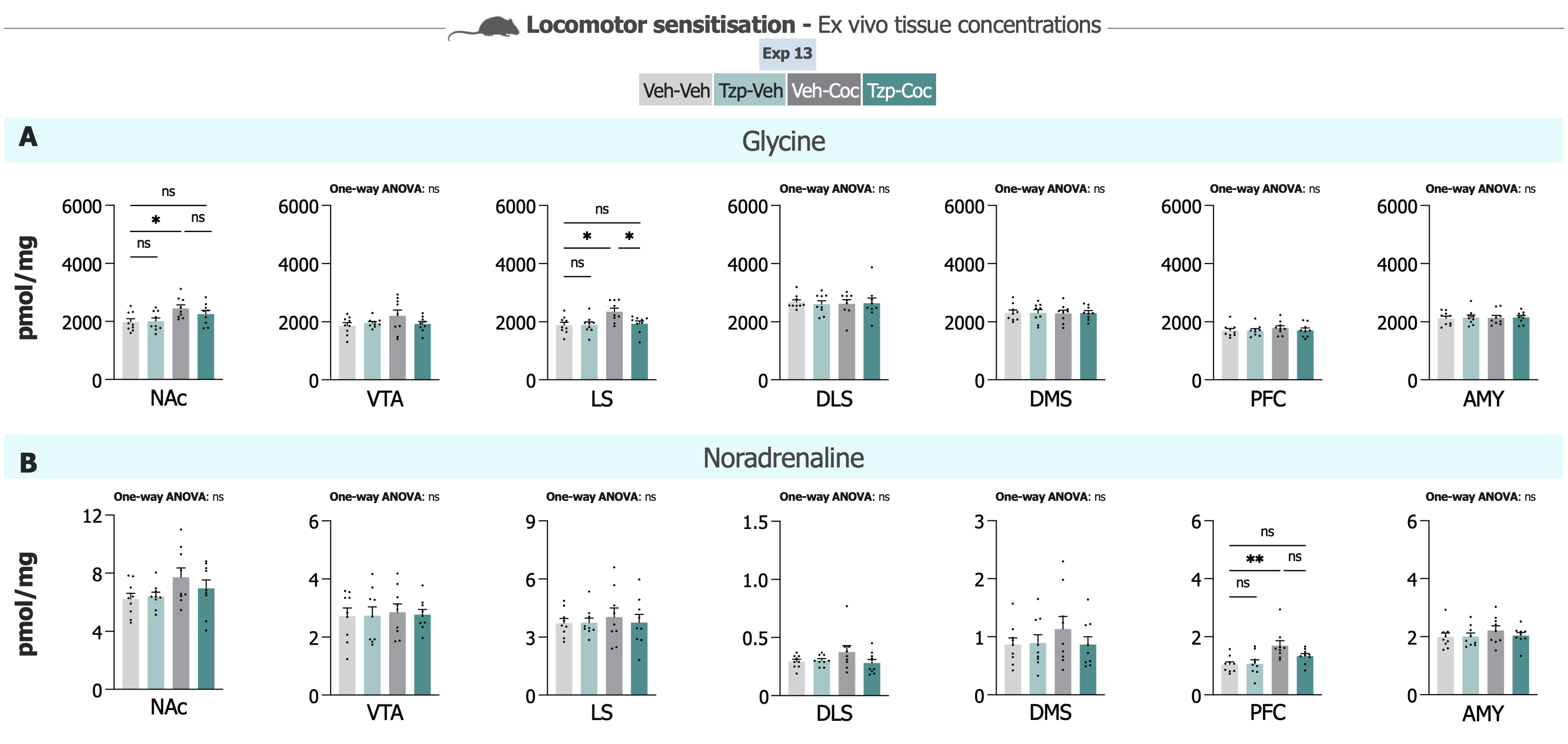
**

**Supplementary Figure 9. Glycine and noradrenaline alterations across brain regions following tirzepatide treatment in cocaine-sensitised mice.**

**A-B.** Ex vivo tissue concentration analysis of glycine and noradrenaline across seven brain regions following repeated cocaine (Coc; 10 mg/kg) or vehicle exposure with (Tzp; 30 nmol/kg) or vehicle treatment in locomotor-sensitised male mice (n=9/group, one-way ANOVA followed by Bonferroni post hoc test). **A.** Glycine concentrations show cocaine-induced increases in the NAc and LS. Tirzepatide normalises elevations in the LS, while NAc alterations remain largely unchanged. **B.** Noradrenaline concentrations demonstrate cocaine-induced increases in the PFC, while other regions remain largely unaffected. Data are presented as mean±SEM. ns; non-significant, *P<0.05, **P<0.01.


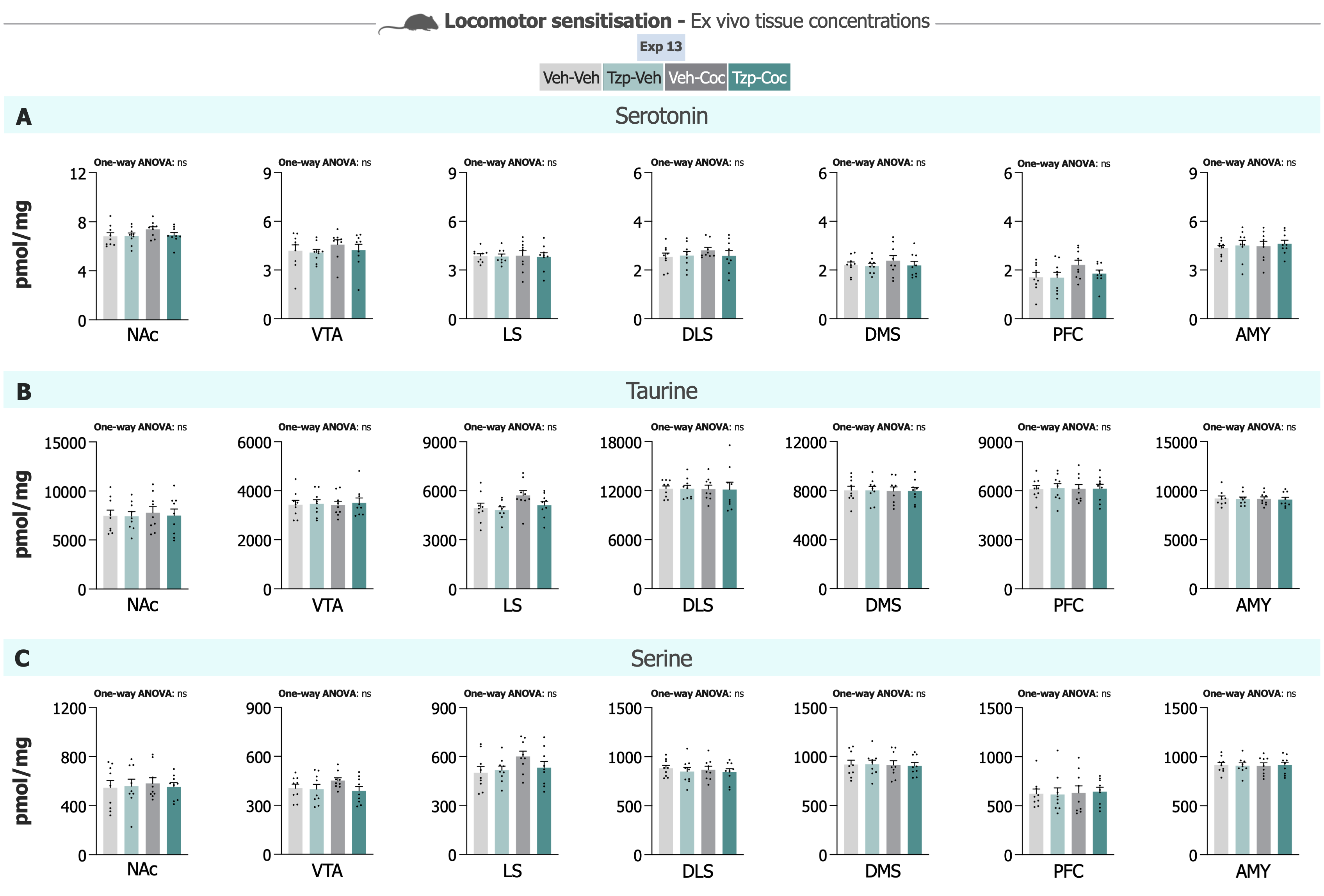
**Supplementary Figure 10**

**Supplementary Figure 10. Serotonin, taurine, and serine alterations across brain regions following tirzepatide treatment in cocaine-sensitised mice.**

**A-C.** Ex vivo tissue concentration analysis of serotonin, taurine, and serine across seven brain regions following repeated cocaine (Coc; 10 mg/kg) or vehicle exposure with tirzepatide (Tzp; 30 nmol/kg) or vehicle treatment in locomotor-sensitised male mice (n=9/group, one-way ANOVA). Minimal alterations to all three neurotransmitters are observed throughout the seven regions analysed. Data are presented as mean±SEM. ns; non-significant.


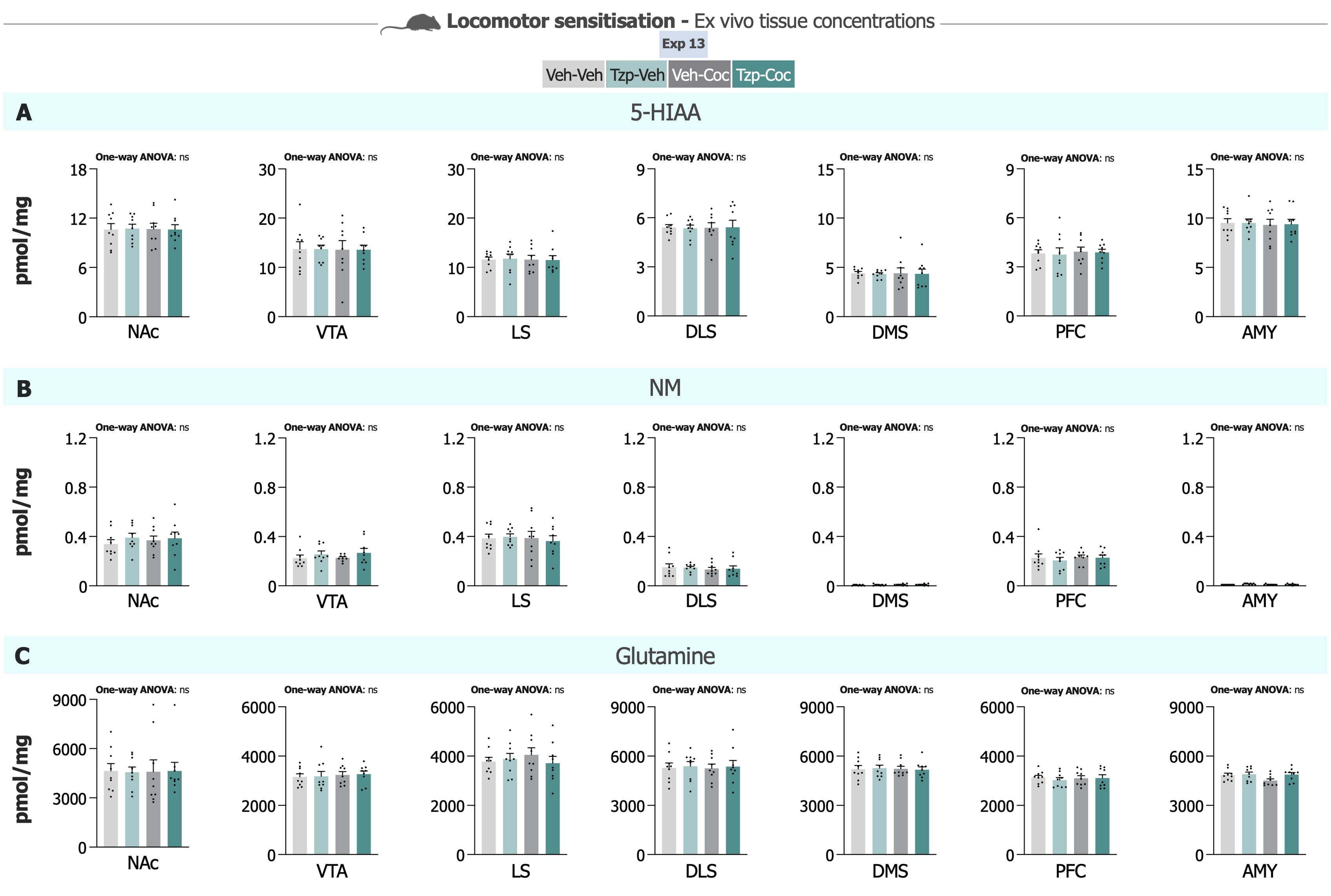
**Supplementary Figure 11**

**Supplementary Figure 11. 5-HIAA, NM and glutamine alterations across brain regions following tirzepatide treatment in cocaine-sensitised mice.**

**A-C.** Ex vivo tissue concentration analysis of 5-HIAA, NM and glutamine across seven brain regions following repeated cocaine (Coc; 10 mg/kg) or vehicle exposure with tirzepatide (Tzp; 30 nmol/kg) or vehicle treatment in locomotor-sensitised male mice (n=9/group, one-way ANOVA). Minimal alterations to all three neurotransmitters are observed throughout the seven regions analysed. Data are presented as mean±SEM. ns; non-significant.


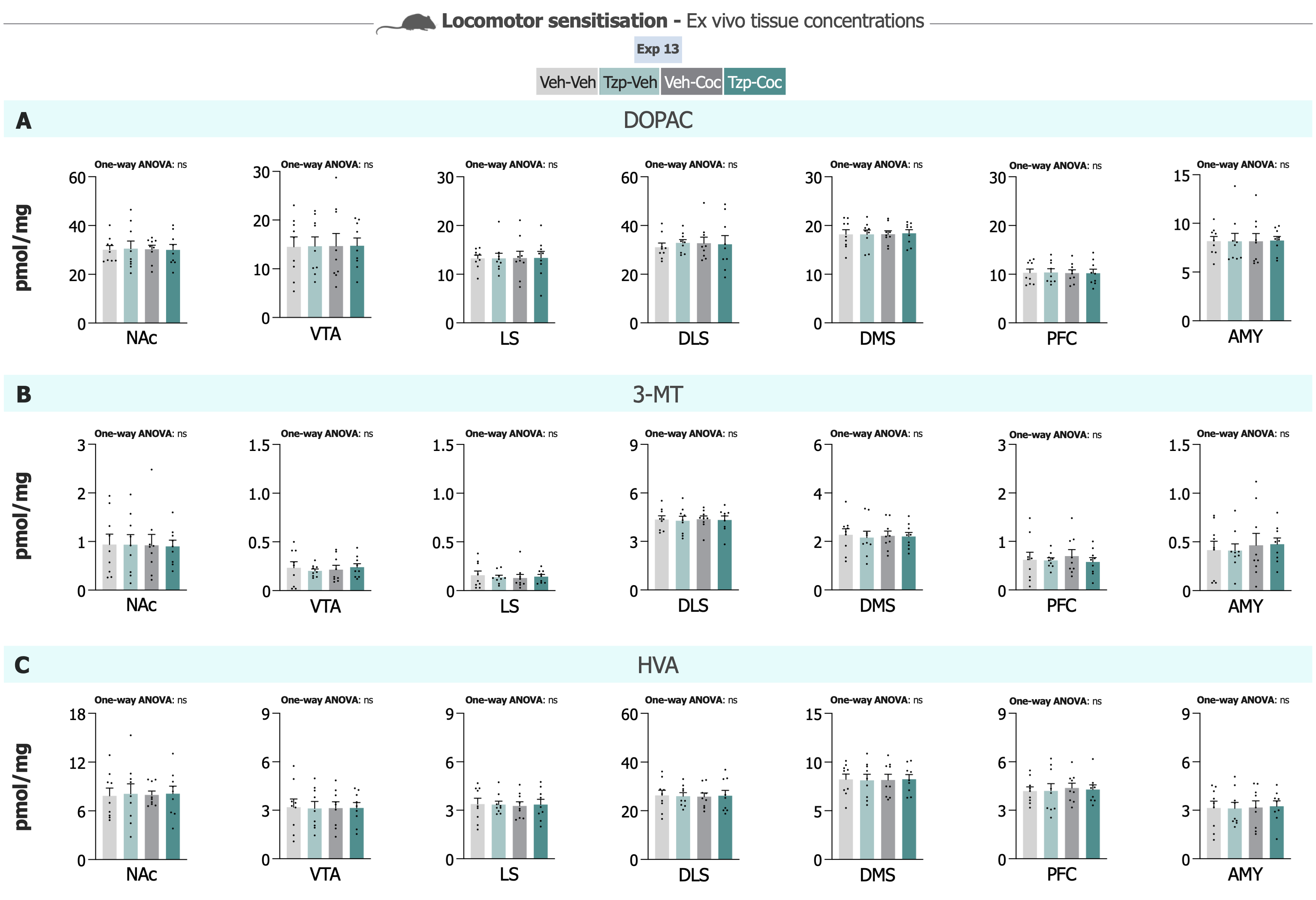
**Supplementary Figure 12**

**Supplementary Figure 12. DOPAC, 3-MT, and HVA alterations across brain regions following tirzepatide treatment in cocaine-sensitised mice.**

**A-C.** Ex vivo tissue concentration analysis of DOPAC, 3-MT, and HVA across seven brain regions following repeated cocaine (Coc; 10 mg/kg) or vehicle exposure with tirzepatide (Tzp; 30 nmol/kg) or vehicle treatment in locomotor-sensitised male mice (n=9/group, one-way ANOVA). Minimal alterations to all three neurotransmitters are observed throughout the seven regions analysed. Data are presented as mean±SEM. ns; non-significant.


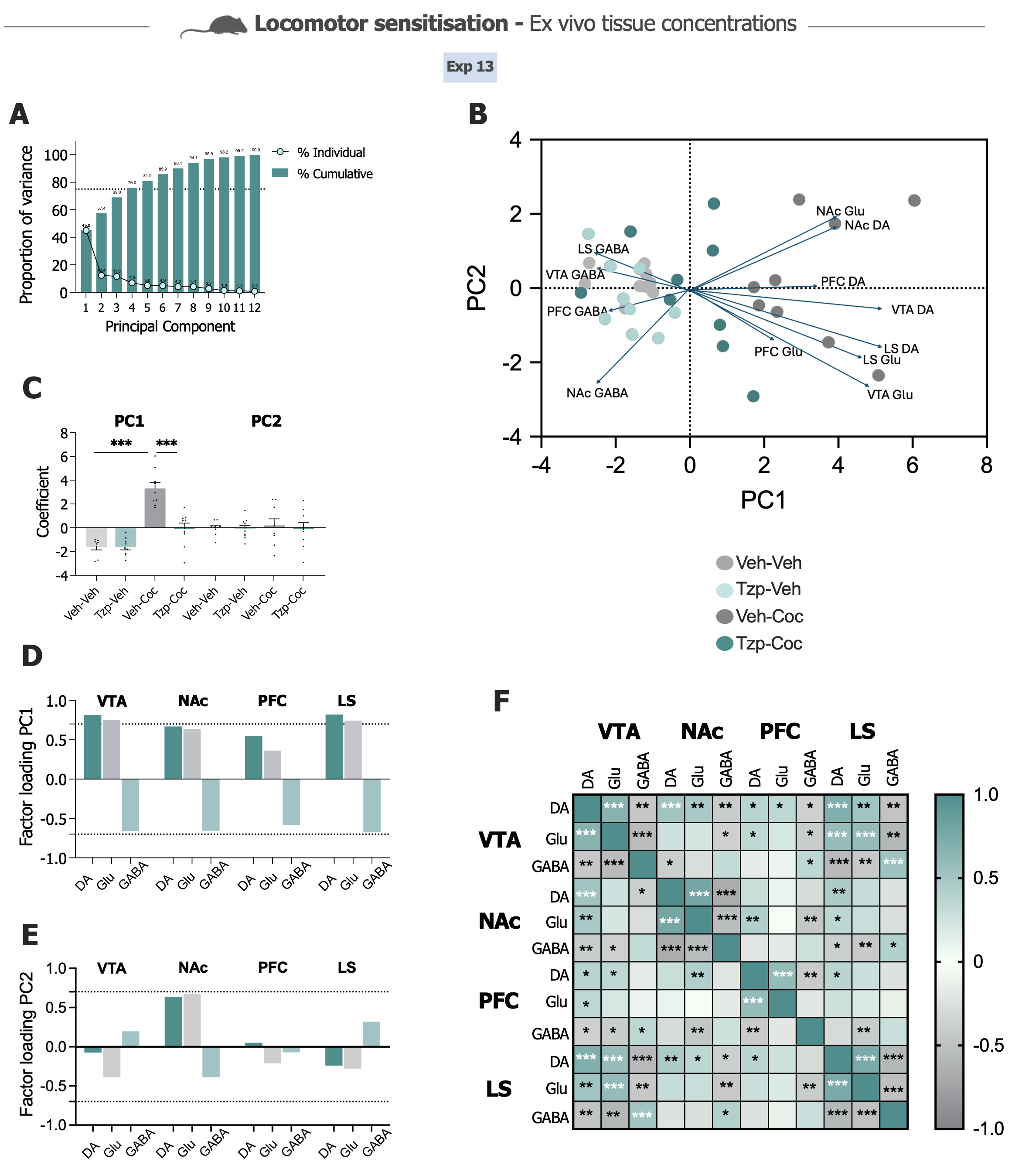
**Supplementary Figure 13**

**Supplementary Figure 13. Principal component analysis reveals neurochemical interdependence across mesocorticolimbic circuits in mice from the locomotor sensitisation experiment.**

**A-F.** Principal component analysis (PCA) across all four treatment groups examined neurochemical interdependence following repeated cocaine (Coc; 10 mg/kg) or vehicle exposure and either tirzepatide (Tzp; 30 nmol/kg) or vehicle treatment in mice from the locomotor sensitisation experiment (n=9/group, one-way ANOVA followed by Bonferroni post hoc test). **A-B.** PC1 explained 45% of total variance, while PC2 explained 12% of total variance. **C.** PC1 effectively discriminated subjects by treatment exposure, while PC2 showed no treatment group differences. **D.** Factor loadings showed GABA presented a negative correlation with PC1, while dopamine (DA) in the VTA (81 %) and LS (82 %) contributed strongly to the variance. Dotted line marks a factor loading of 0.7. **E.** PC2 accounted for 12% of variance, with NAc glutamate (Glu) as the main contributor (67 %). **F.** Correlation matrix based on Pearson correlation coefficients demonstrated that VTA dopamine correlated significantly with all analysed neurotransmitters and brain regions, revealing strong interregional associations. Data are presented as mean±SEM. ns; non-significant, *P<0.05, **P<0.01, ***P<0.001.


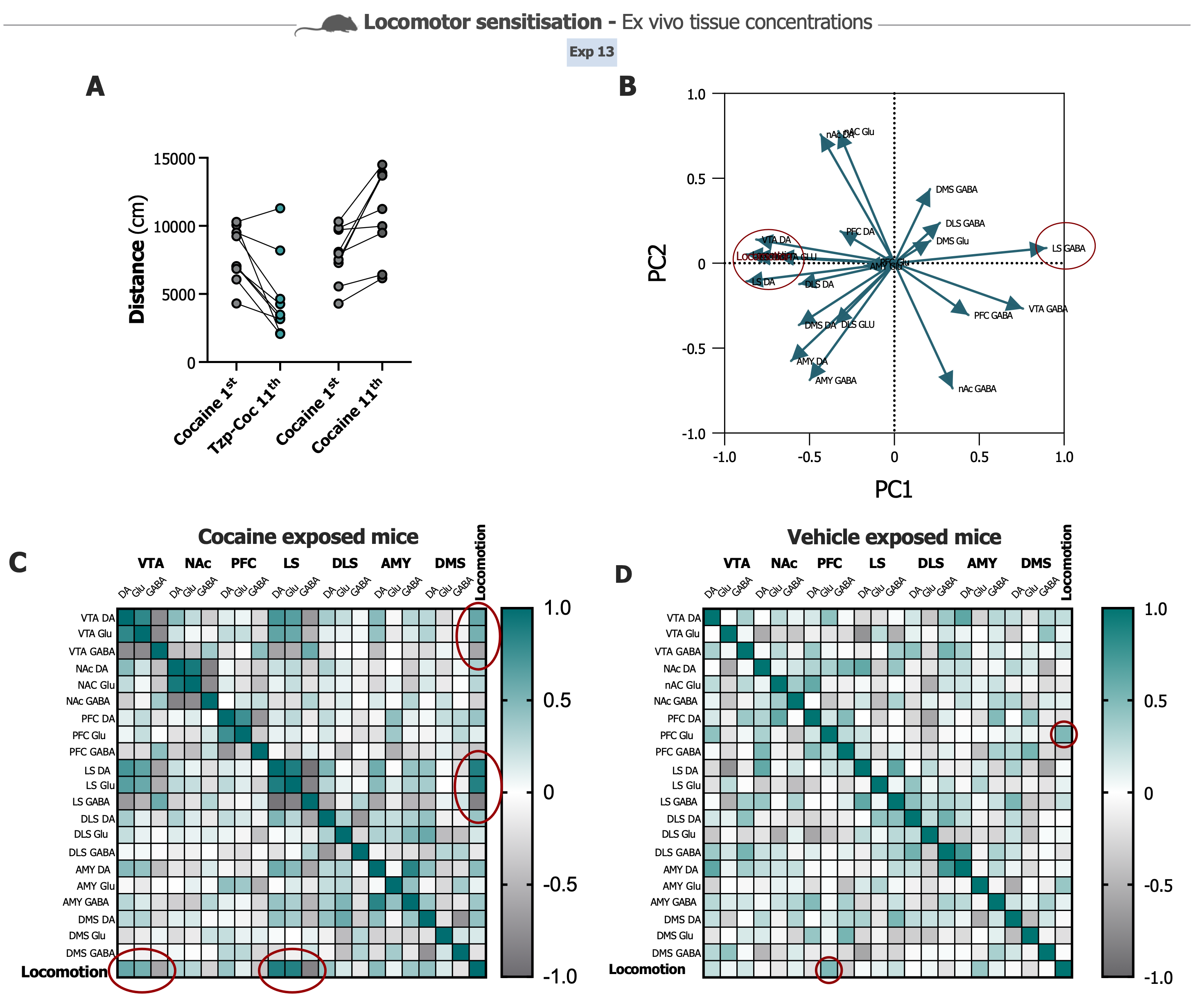
**Supplementary Figure 14**

**Supplementary Figure 14. Principal component analysis including locomotion reveals neurochemical interdependence across mesocorticolimbic circuits in mice from the locomotor sensitisation experiment.**

**A.** Locomotor activity elicited by repeated exposure to cocaine was significantly depressed by tirzepatide, but interindividual variability was observed. **B.** Principal component analysis (PCA) confirmed that factor loading for locomotion completely overlapped with glutamate (Glu) in LS and VTA with dopamine (DA) projecting in the same direction, and LS GABA projected in the opposite. **C.** Correlation analysis of neurochemistry in defined brain regions and cocaine-induced locomotor behaviour demonstrated an association between distance travelled and VTA and LS, where the correlation with neurochemistry in LS was especially strong. **D.** For vehicle exposed mice, the distance travelled was not associated with neurochemistry in LS, but significantly correlated with glutamate levels in PFC.
